# Visual imaging as a predictor of neurodegeneration in experimental autoimmune demyelination and multiple sclerosis

**DOI:** 10.1186/s40478-022-01391-y

**Published:** 2022-06-15

**Authors:** Gabrielle M. Mey, Kirsten S. Evonuk, McKenzie K. Chappell, Laura M. Wolfe, Rupesh Singh, Julia C. Batoki, Minzhong Yu, Neal S. Peachey, Bela Anand-Apte, Robert Bermel, Daniel Ontaneda, Kunio Nakamura, Kedar R. Mahajan, Tara M. DeSilva

**Affiliations:** 1 Department of Neurosciences, Lerner Research Institute, Cleveland Clinic Foundation, and Case Western Reserve University, 9500 Euclid Avenue, Cleveland, OH 44195 USA; 2 Cole Eye Institute, Cleveland Clinic Foundation, Cleveland, OH USA; 3 Department of Ophthalmology, Cleveland Clinic Lerner College of Medicine of Case Western Reserve University, Cleveland, OH USA; 4 Louis Stokes Cleveland VA Medical Center, Cleveland, OH USA; 5 Mellen Center for MS Treatment and Research, Neurological Institute, Cleveland Clinic Foundation, Cleveland, OH USA; 6 Department of Biomedical Engineering, Lerner Research Institute, Cleveland Clinic Foundation, Cleveland, OH USA; 7 Present Address: Hooke Laboratories, Inc., Lawrence, MA USA

## Abstract

Thalamic volume is associated with clinical disability in multiple sclerosis (MS) and is vulnerable to secondary neurodegeneration due to its extensive connectivity throughout the central nervous system (CNS). Using a model of autoimmune demyelination that exhibits CNS-infiltrating immune cells in both spinal cord white matter and optic nerve, we sought to evaluate neurodegenerative changes due to lesions affecting the spino- and retino-thalamic pathways. We found comparable axonal loss in spinal cord white matter and optic nerve during the acute phase of disease consistent with synaptic loss, but not neuronal cell body loss in the thalamic nuclei that receive input from these discrete pathways. Loss of spinal cord neurons or retinal ganglion cells retrograde to their respective axons was not observed until the chronic phase of disease, where optical coherence tomography (OCT) documented reduced inner retinal thickness. In patients with relapsing–remitting MS without a history of optic neuritis, OCT measures of inner retinal volume correlated with retino-thalamic (lateral geniculate nucleus) and spino-thalamic (ventral posterior nucleus) volume as well as neuroperformance measures. These data suggest retinal imaging may serve as an important noninvasive predictor of neurodegeneration in MS.

**Supplementary Information:**

The online version contains supplementary material available at 10.1186/s40478-022-01391-y.

Multiple sclerosis (MS) is a chronic inflammatory demyelinating and neurodegenerative disease of the central nervous system (CNS) initially presenting with T2-weighted white matter lesions by magnetic resonance imaging (MRI). Although immunomodulatory therapies reduce relapse rate and improve quality of life, MS is a progressive neurodegenerative disease resulting in permanent disability for which there is no remedy [1, 2]. Early detection of MS and MS progression is critical to facilitate the most effective use of current and future therapies. Towards this goal, several studies have demonstrated that gray matter atrophy occurs early in the MS disease course and is a predictor of clinical disability, with thalamic atrophy being one of the strongest indicators of worsening disability [3–6]. Thus, understanding the spatio-temporal mechanisms that drive neurodegeneration in MS is an important unmet need for the development of neuroprotective strategies. Histopathology and MRI of the thalamus in postmortem MS brains support that thalamic volume correlates better with damage in extra-thalamic regions than with lesions directly in the thalamus [7]. Effectiveness of disease-modifying therapies has been associated with reduced thalamic loss [8] and clinical trials utilize thalamic atrophy as an outcome measure for neurodegeneration [9–11], emphasizing the need to understand damage to pathways that innervate the thalamus.

The thalamus receives presynaptic input from axons that arise from white matter structures, making the study of its afferent and efferent projections important to advance our understanding of neurodegeneration in MS. In this regard, the most commonly used preclinical model to test therapeutics for the treatment of MS was utilized, the C57BL/6J mouse model of chronic autoimmune demyelination, referred to as experimental autoimmune encephalomyelitis (EAE) [12]. The advantage of using this model is that CNS-infiltrating myelin–reactive T cells that initiate autoimmune demyelination are predominantly found in the spinal cord (SC) white matter and optic nerve and are relatively non-existent in the cerebrum. This affords the unique opportunity to elucidate the spatio-temporal relationship between neuroinflammation and neurodegeneration in white versus gray matter regions comprising the spino- and retino-thalamic pathways *independent* of CNS-infiltrating immune cells in the forebrain that could impact thalamic pathology.

In addition to MRI measures of thalamic atrophy, non-invasive visual imaging assessments are becoming an important correlate of MS disability. Thinning of the retinal nerve fiber (RNFL) and ganglion cell/inner plexiform layers (GC/IPL) of the retina documented by noninvasive optical coherence tomography (OCT) imaging have been associated with brain atrophy and white matter damage in MS [13–15]. Retinal thinning occurs in MS in the absence of overt clinical optic neuritis and is associated with impaired visual function [16–19]. The molecular underpinnings of visual changes in MS and how they relate to damage in the rest of the CNS remain to be determined. Here, the use of EAE allows for the investigation of histopathological changes that occur alongside structural and functional visual assessments. Retinal ganglion cell (RGC) loss and thinning of inner retinal layers measured by OCT correlate with disability scores and are accompanied by reactive gliosis in the optic nerve and retina during EAE [20–27]. However, the spatio-temporal relationship of demyelination, axonal loss, and neurodegeneration in the spino- and retino-thalamic pathways and how these pathological changes translate to structural and functional visual deficits is not clear. Timing delays in the visual evoked potential (VEP) are a well-established feature of both MS [28–30] and EAE [27, 31, 32]. In the EAE model, we show that VEP delays coincided with acute loss of myelinated axons in the optic nerve and SC white matter while OCT imaging tracked retinal thinning consistent with loss of RGCs and SC ventral horn neurons during chronic EAE. To determine the relevance of this model to changes in the spino- and retino-thalamic pathways in MS, OCT measures of inner retinal volume in patients with MS who had no history of optic neuritis were associated with MRI measures of retino-thalamic (lateral geniculate) and spino-thalamic (ventral posterior) regions of the thalamus, which correlated with neuroperformance measures.

## Results

### Inflammation, demyelination, and axonal loss occur similarly in the spinal cord white matter and optic nerve

**Fig. 1:**
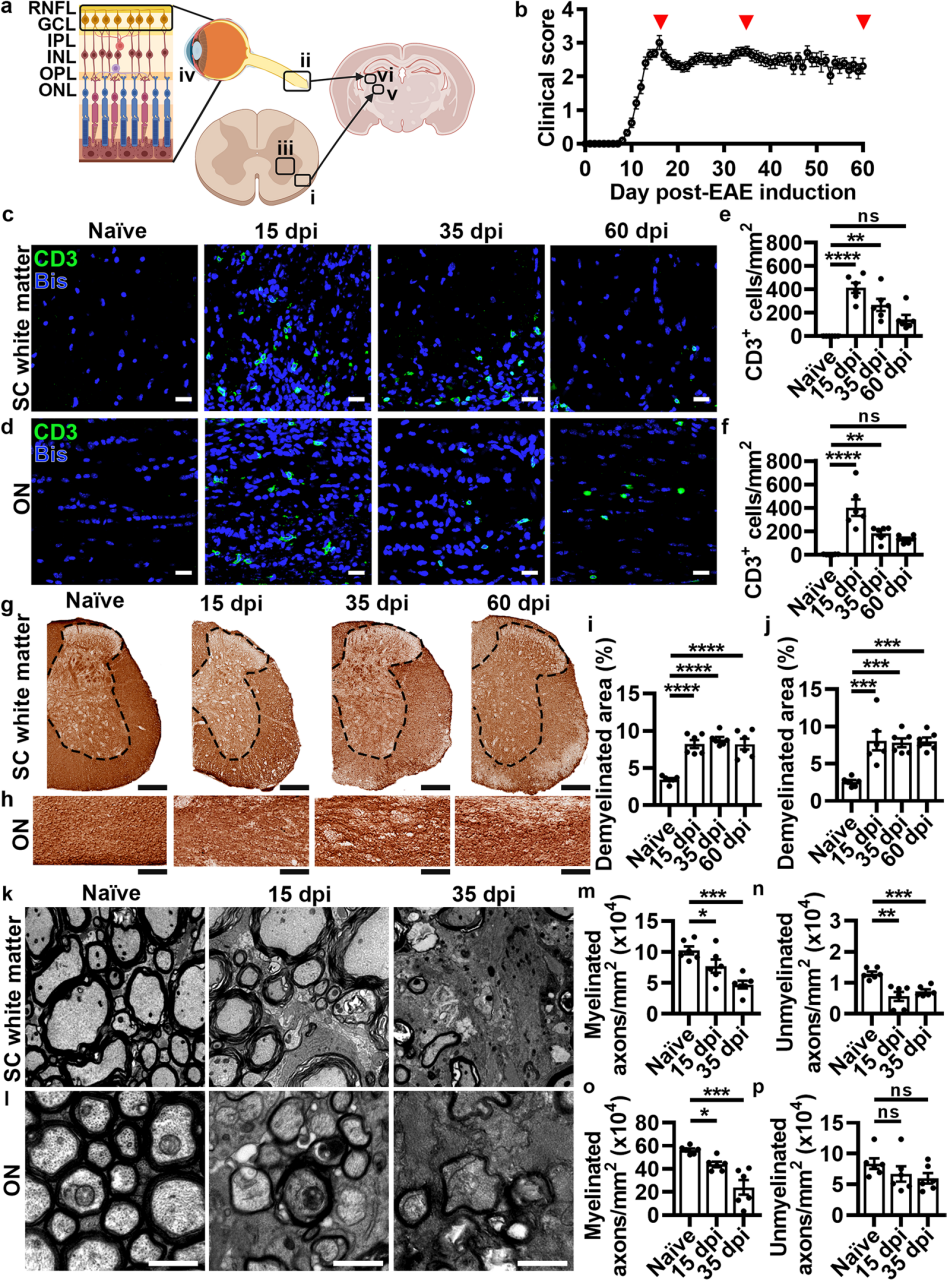
T cell infiltration and myelin/axonal pathology follow similar time courses in spinal cord white matter and optic nerve. (**a**) Schematic of neural pathways in the spinal cord (SC) white matter (**a**_**i**_), optic nerve (ON) (**a**_**ii**_), SC gray matter (**a**_**iii**_), retina (**a**_**iv**_), and ventral posterolateral (VPL) (**a**_**v**_), and dorsal lateral geniculate (dLGN) (**a**_**vi**_) nuclei of the thalamus. (**b**) EAE clinical scores. Analyses were performed at 15 (peak disease), 35 (chronic disease), and 60 dpi (sustained chronic disease), indicated by red arrowheads. *P* values listed consecutively are naïve vs 15, 35, or 60 dpi, respectively. (**c-d**) Representative images of CD3^+^ T cells in the SC white matter and ON. Scale bars = 20 µm. (**e–f**) Quantification of CD3^+^ T cells (**e**, SC: ****P < 0.0001, **P = 0.0017, *P* = 0.0745; **f**, ON: ****P < 0.0001, **P = 0.0092,* P* = 0.0502). (**g-h**) Representative images of 3,3'-diaminobenzidine (DAB) staining for myelin basic protein (MBP) in the SC white matter (gray matter of SC within black dotted line, white matter for analysis outside dotted line) and ON. Scale bars = 100 µm. (**i-j**) Quantification of demyelinated area (**i**, SC: ****P < 0.0001, ****P < 0.0001, ****P < 0.0001; **j**, ON: ***P = 0.0003, ***P = 0.0003, ***P = 0.0003). (**k-l**) Representative electron micrographs (EM) in the SC white matter and ON. Scale bars = 5 µm (SC) and 1 µm (ON). (**m-p**) Quantification of myelinated (**m**, SC: *P = 0.0425, ***P = 0.0006; **o**, ON: *P = 0.0433, ***P = 0.0001) and unmyelinated (**n**, SC: ***P = 0.0004, **P = 0.0023; **p**, ON: *P* = 0.4455, *P* = 0.3026) axons (see Additional file 1: Figure S1c-d for total axon counts). Statistical differences were determined by one-way analysis of variance (ANOVA) with Holm-Šidák post-hoc test. All data are expressed as means ± SEM, *n* = 6 mice per group, 12–16 fields from 3–4 sections (CD3, MBP) or 5–6 fields (EM) per mouse. Bis = bisbenzimide

The time course of T cell infiltration, demyelination, axonal loss, and reactive gliosis during EAE was evaluated in the ventral and lateral funiculi of the lumbar SC white matter (Fig. 1a_i_), which is where lesions are typically studied in this model, in comparison to the optic nerve (Fig. 1a_ii_). Neuronal cell body loss retrograde to SC white matter and optic nerve, which includes SC ventral horn neurons (Fig. 1a_iii_) and RGCs within the ganglion cell layer (GCL) in the retina (Fig. 1a_iv_), respectively, was also investigated. Although the SC ventral white matter contains several different ascending and descending axonal projections, assessing ventral horn neuron loss provides insight into the SC-driven motor phenotype (i.e. EAE clinical scores). The visual system, however, provides a clear substrate to evaluate axonal damage in relation to its respective cell body as all optic nerve axons arise from RGCs. Therefore ventral horn neuron and RGC loss were compared to establish whether retrograde neuronal loss contributes to EAE pathology in both the SC and visual system. T cell infiltration and reactive gliosis were also assessed in the SC ventral horn (Fig. 1a_iii_) and layers of the retina including the RNFL (unmyelinated axons of RGCs that exit the lamina cribrosa and are myelinated to form the optic nerve), GCL, inner plexiform layer (IPL, comprising synapses between amacrine, bipolar cells, and RGCs), inner nuclear layer (INL, housing the cell bodies of amacrine and bipolar cells), outer plexiform layer (OPL, containing synapses between photoreceptors and second-order neurons) and outer nuclear layer (ONL, housing photoreceptor cell bodies) (Fig. 1a_iv_). Neuronal loss was evaluated in the ventral posterolateral nucleus (VPL, Fig. 1a_v_), which receives synaptic input from axons in SC white matter, and the dorsal lateral geniculate nucleus (dLGN, Fig. 1a_vi_), which receives synaptic input from optic nerve axons.

Assessments were performed at three time points including peak of disease (15 days post-EAE induction (dpi)), chronic disease (35 dpi), and sustained chronic disease (60 dpi; Fig. 1b, red arrowheads). The initiating event in EAE is myelin-reactive T cell infiltration into the white matter after induction with myelin oligodendrocyte glycoprotein (MOG), followed by demyelination and axonal injury. T cells were identified by immunofluorescent staining for CD3 and colocalized with bisbenzamide (Bis), a marker of cell nuclei, in naïve (untreated) mice and at 15, 35, and 60 dpi in the SC white matter (Fig. 1c) and optic nerve (Fig. 1d). CD3^+^ T cells were significantly increased in EAE mice compared to naïve controls at 15 and 35 dpi in the SC white matter (Fig. 1e) and optic nerve (Fig. 1f). By 60 dpi this response had dissipated (Fig. 1e–f). Across all time points there was a significant linear relationship between the numbers of infiltrating CD3^+^ T cells in SC white matter and optic nerve (Additional file 1: Figure S1a). Demyelination in these regions was quantified using 3, 3'-diaminobenzidine (DAB) to stain for anti-myelin basic protein (MBP), measured as the unstained area fraction of MBP in the SC white matter (Fig. 1g, region outside the dotted line) and optic nerve (Fig. 1h). A statistically significant increase in demyelinated area was observed at 15 dpi in both SC white matter (Fig. 1i) and optic nerve (Fig. 1j) compared to naïve mice that persisted at 35 and 60 dpi. Similar to CD3^+^ infiltrating T cells, there was a significant linear relationship between demyelination in the SC white matter and optic nerve across all time points (Additional file 1: Figure S1b).

To determine if axonal injury coincided with the timing of demyelination, ultrastructural analysis of the SC white matter (Fig. 1k) and optic nerve (Fig. 1l) using electron microscopy was performed. A statistically significant reduction in myelinated axons was observed at 15 and 35 dpi in the SC white matter (Fig. 1m) and optic nerve (Fig. 1o), consistent with loss of myelin (Fig. 1g–j). Unmyelinated axons were also significantly decreased in the SC white matter (Fig. 1n). In the optic nerve a decreased, but not significant, loss of unmyelinated axons was observed (Fig. 1p), likely driven by the relatively dense compaction of myelinated axons compared to the ventral and lateral funiculi in the SC. Nonetheless, the total number of axons for both SC white matter (Additional file 1: Figure S1c) and optic nerve (Additional file 1: Figure S1d) was significantly decreased and exhibited a significant linear relationship across all EAE time points (Additional file 1: Figure S1e). Percentage of myelinated and unmyelinated axons normalized to naïve total axons is shown Additional file 1: Figure S1f-i. To ensure that age-related differences were not confounding the quantitative analyses, age-matched naïve controls for 15, 35, and/or 60 dpi were compared. No age-related differences were observed for CD3^+^ T cells in the SC white matter (Additional file 1: Figure S1j) and optic nerve (Additional file 1: Figure S1k), demyelinated area by MBP immunostaining in SC white matter (Additional file 1: Figure S1l) and optic nerve (Additional file 1: Figure S1m), or total axon counts in electron micrographs in SC white matter (Additional file 1: Figure S1n) and optic nerve (Additional file 1: Figure S1o). Therefore, EAE time points were compared to the naïve cohort matched to the oldest age group, which was 60 dpi for immunohistochemical analyses, and 35 dpi for axon counts by electron microscopy. Overall, these data provide evidence that T cells infiltrate the SC white matter and optic nerve similarly during EAE, leading to myelin and axonal injury in both regions and suggesting concurrent spatio-temporal time courses for autoimmune demyelination.

The extent of reactive gliosis was evaluated by calculating the mean area fraction of GFAP immunofluorescent staining for astrocytes and of Iba1 staining for microglia/macrophages in the SC white matter (Additional file 1: Figure S2a,c) and optic nerve (Additional file 1: Figure S2b,d). At 15 dpi, a statistically significant increase in GFAP^+^ labeling was observed in both the SC white matter (Additional file 1: Figure S2e) and optic nerve (Additional file 1: Figure S2f), which remained significantly elevated at 35 and 60 dpi in comparison to naïve controls. A similar pattern was observed for Iba1 staining with a significant increase in the SC white matter (Additional file 1: Figure S2g) and optic nerve (Additional file 1: Figure S2h) at 15 dpi that persisted at 35 and 60 dpi. Analogous to CNS infiltration of T cells, demyelination, and axonal loss, there was a significant correlation for reactive gliosis during all EAE time points between the SC white matter and optic nerve (Additional file 1: Figure S1p-q). A low-powered view of SC white matter (Figure S3a) and optic nerve (Additional file 1: Figure S3b) demonstrates focal areas of increased CD3^+^ T cells at 15 and 35 dpi (quantified in Fig. 1e-f), with an enlarged area of Iba1 immunoreactivity (quantified in Additional file 1: Figure S2g-h) and more Bis^+^ nuclei. In SC white matter and optic nerve at 60 dpi, however, focal infiltrates of CD3^+^ T cells (Additional file 1: Figure S3a–b) were decreased (as quantified in Fig. 1e–f), yet there is a sustained increase in Iba1 immunoreactivity (Additional file 1: Figure S3a-b, quantified in Additional file 1: Figure S2g–h). These data provide evidence that after CNS infiltrating CD3^+^ T cells are diminished, glial cells remain activated in both SC white matter and optic nerve. High-powered images of chronic EAE lesions at 35 dpi demonstrate concurrent infiltrating CD3^+^ T cells in the SC white matter (Additional file 1: Figure S4a) and optic nerve (Additional file 1: Figure S4b) along with loss of MBP (quantified in Fig. 1i–j) and increased Iba1^+^ area (quantified in Additional file 1: Figure S2g–h) and Bis^+^ nuclei.

### Retrograde loss of ventral horn neurons and RGCs following damage to their respective axons

**Fig. 2:**
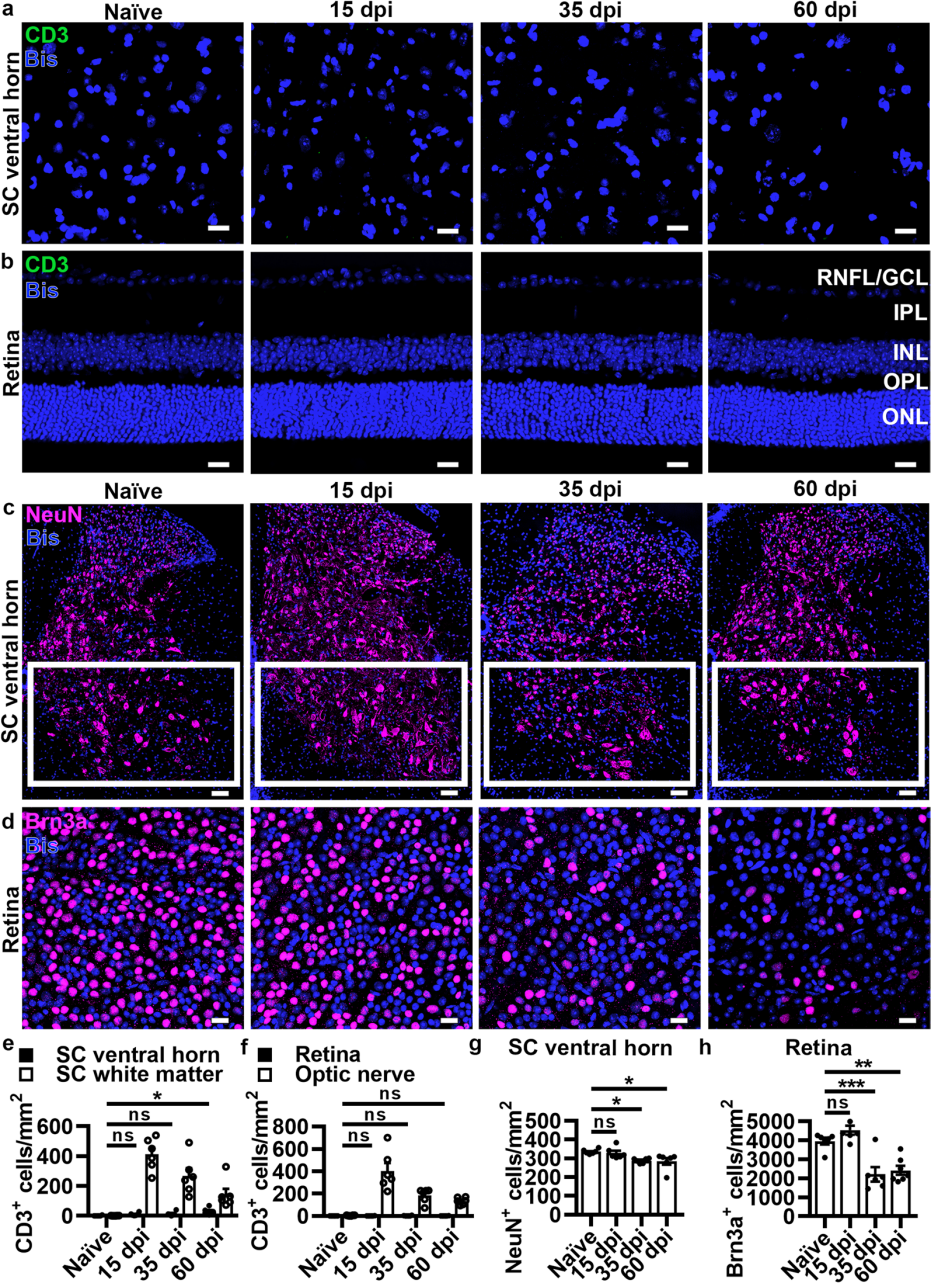
Neuronal cell loss occurs in the spinal cord ventral horn and retina during chronic EAE. (**a-b**) Representative images of CD3^+^ T cells in the SC ventral horn and retina with retinal layers labeled. Scale bars = 20 µm. (**c-d**) Representative images of NeuN^+^ cells in the SC ventral horn and Brn3a^+^ RGCs in the retina. Scale bars = 100 µm (SC) and 20 µm (retina). *P* values listed consecutively are naïve vs 15, 35, or 60 dpi, respectively. (**e–f**) Quantification of CD3^+^ cells in SC ventral horn relative to SC white matter (**e**, SC ventral horn compared to naïve: *P* = 0.5095, *P* = 0.4159, *P = 0.0173; SC ventral horn compared to SC white matter naïve: *P* = 0.9997, 15 dpi: *P* < 0.0001, 35 dpi: *P* < 0.0001, 60 dpi: *P* = 0.0666) and CD3^+^ T cells in the retina relative to ON (**f**, retina compared to naïve: *P* = 0.8109, *P* = 0.4692, *P* = 0.8611; retina compared to optic nerve naïve: *P* > 0.9999, 15 dpi: *P* < 0.0001, 35 dpi: *P* = 0.0004, 60 dpi: *P* = 0.0171). (**g-h**) Quantification of NeuN^+^ cells in SC ventral horn (region outlined in white boxes, *P* = 0.8769, *P = 0.0405, *P = 0.0286) and Brn3a^+^ cells in the retina (*P* = 0.2820, ***P = 0.0007, **P = 0.0014). Statistical differences were determined by one or two-way ANOVA with Holm-Šidák post-hoc test. All data are expressed as means ± SEM including *n* = 6 mice per group, 4–6 fields from 2–3 sections (SC), 12–16 fields from 3–4 sections (retina CD3) and 12 fields from one whole retina (Brn3a) per mouse. Bis = bisbenzimide

After establishing a similar time course for inflammation, demyelination, and axonal loss in SC white matter and optic nerve, neuronal cell body loss retrograde to their respective axons in the SC white matter and optic nerve was assessed. In the SC, neuronal cell bodies retrograde to axons in the ventral white matter (Fig. 1a_i_) are found in the ventral horn (Fig. 1a_iii_). In the visual system, neuronal cell bodies retrograde to axons in the optic nerve (Fig. 1a_ii_) are RGCs located in the GCL (Fig. 1a_iv_). CD3^+^ T cells colocalized with Bis were quantified in the SC ventral horn (Fig. 2a) and retina including the following layers: RNFL/GCL, IPL, INL, OPL, and ONL (Fig. 2b). CD3^+^ T cells did not significantly infiltrate the SC ventral horn (Fig. 2e, SC white matter shown for comparison) or the retina (Fig. 2f, optic nerve shown for comparison) at any EAE time point with the exception of a slight increase at 60 dpi in the SC ventral horn. Reactive gliosis in the SC ventral horn was assessed by GFAP^+^ (Additional file 1: Figure S5a) and Iba1^+^ (Additional file 1: Figure S5j) area, which was significantly elevated by 15 dpi (Additional file 1: Figure S5c and S5l, respectively) and sustained through 60 dpi similar to its adjacent white matter (Additional file 1: Figure S2e and S2g, respectively). In the retina, however, a significant increase in GFAP^+^ area (Additional file 1: Figure S5b) for all layers combined was not observed until 35 dpi (Additional file 1: Figure S5d). Assessment of each discrete layer of the retina revealed that the statistical significance for GFAP immunoreactivity was driven by the RNFL/GCL (Additional file 1: Figure S5e) and the IPL (Additional file 1: Figure S5f), but no significant change was observed in the INL, OPL, or ONL (Additional file 1: Figure S5g–i). Similarly, quantification of Iba1^+^ immunostaining for combined layers of the retina (Additional file 1: Figure S5k) was not statistically increased until 35 dpi (Additional file 1: Figure S5m). When retinal layers were evaluated separately, this significant increase was observed in the RNFL/GCL, IPL, INL, and OPL (Additional file 1: Figure S5n-q) with no significant change at any time point in the ONL (Additional file 1: Figure S5r).

Neurons were visualized in the SC ventral horn by immunostaining for the neuronal nuclei marker NeuN colocalized with Bis (Fig. 2c, white boxes), whereas in the retina Brn3a, a specific marker for RGCs, was used for immunostaining whole retinas colocalized with Bis (Fig. 2d). Statistical loss of NeuN^+^ neurons did not occur until 35 dpi with a sustained reduction at 60 dpi (Fig. 2g). Likewise, Brn3a^+^ RGCs were not significantly decreased until 35 dpi and sustained at 60 dpi (Fig. 2h) with a significant linear relationship between the number of neurons in the SC ventral horn and RGCs in the retina across all EAE time points (Additional file 1: Figure S6a). Quantification of age-matched naïve controls for 15, 35, and 60 dpi showed no age-related changes for T cell infiltration in the SC ventral horn (Additional file 1: Figure S6b) and retina (Additional file 1: Figure S6c), or for NeuN^+^ cells in the SC ventral horn (Additional file 1: Figure S6d) and Brn3a^+^ cells in the retina (Additional file 1: Figure S6e).

### Thalamic regions that receive synaptic input from the optic nerve and SC white matter do not exhibit neuronal cell body loss, but synaptic loss in the dLGN is observed during acute EAE

Following observations in the SC ventral horn and retina (gray matter regions housing neuronal cell bodies retrograde to white matter axons that sustain injury during EAE), pathology in thalamic regions receiving input from axons in damaged white matter regions was explored. These include the VPL (Fig. 1a_v_), which receives synaptic input from the SC, and the dLGN (Fig. 1a_vi_), which receives input from optic nerve axons. CD3^+^ T cells colocalized with Bis were quantified in the VPL (Additional file 1: Figure S7a) and dLGN (Figure S7b). There was no statistically significant increase in CNS-infiltrating T cells at 15, 35, or 60 dpi compared to naïve controls in the VPL (Figure S7c, SC white matter shown for comparison) or dLGN (Figure S7d, optic nerve shown for comparison). Reactive astrocytosis was evaluated by GFAP^+^ immunostaining in the VPL (Additional file 1: Figure S7e) and dLGN (Additional file 1: Figure S7f). A significant increase in GFAP^+^ area was observed in the VPL at 15 dpi, but it was not sustained at 35 and 60 dpi (Additional file 1: Figure S7g). In the dLGN, however, a significant increase in GFAP^+^ are was observed at 15, 35, and 60 dpi (Additional file 1: Figure S7h). Iba1 was also explored in the VPL (Additional file 1: Figure S7i) and dLGN (Additional file 1: Figure S7j). There was no increase in Iba1^+^ area in the VPL (Additional file 1: Figure S7k) as opposed to the dLGN, where there was a statistically significant increase in Iba1 immunostaining at 15 and 35 dpi (Additional file 1: Figure S7l).

**Fig. 3:**
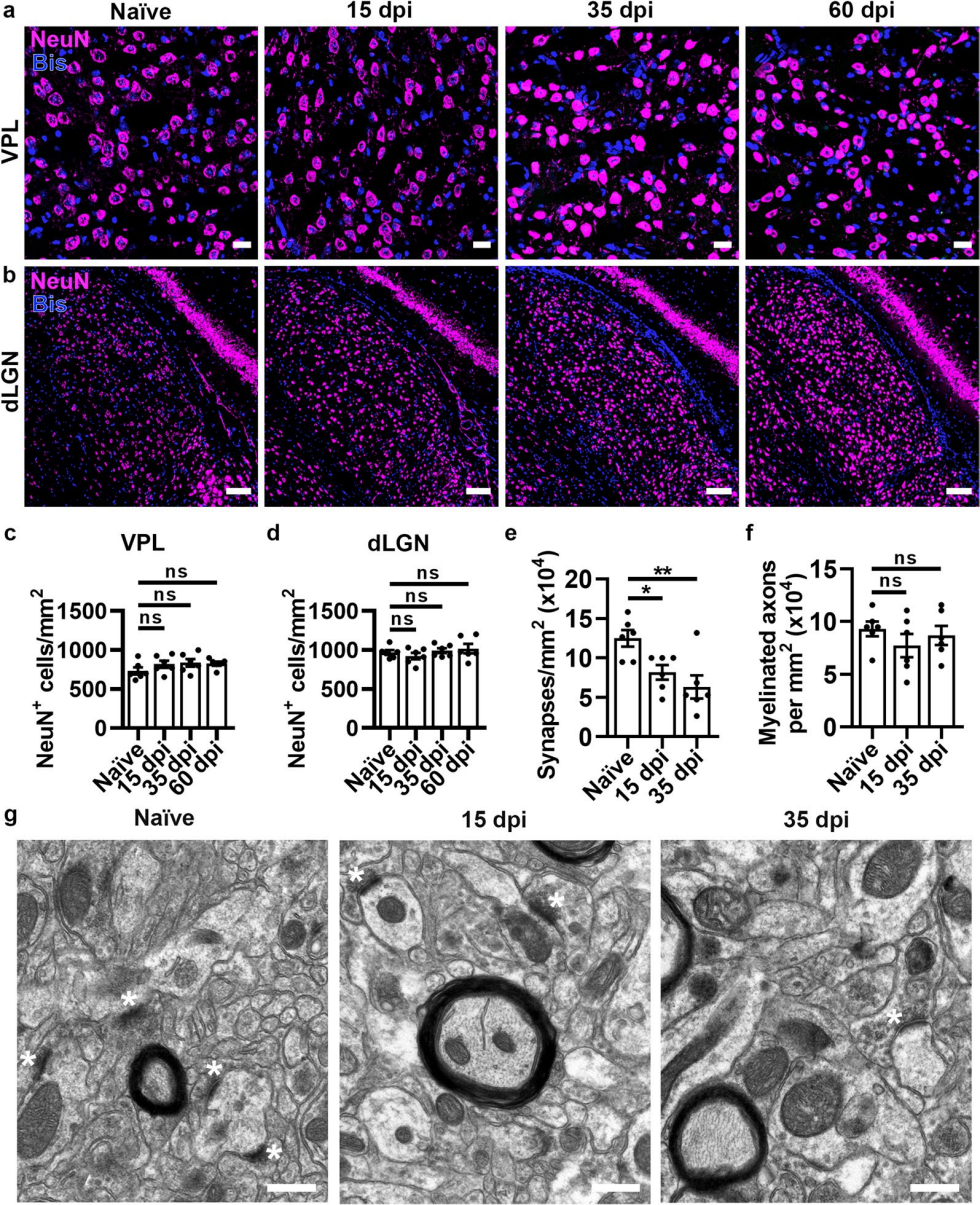
There is no loss of neuronal cell bodies in the VPL and dLGN, but synaptic loss in the dLGN during EAE. (**a-b**) Representative images of NeuN^+^ cells in the VPL and dLGN. Scale bars = 20 µm (VPL) and 100 µm (dLGN)**.** *P* values listed consecutively are naïve vs 15, 35, or 60 dpi, respectively. (**c-d**) Quantification of NeuN^+^ cells (**c**, VPL: *P* = 0.5266, *P* = 0.4775, *P* = 0.5244; **d**, dLGN: *P* = 0.8869, *P* = 0.8869, *P* = 0.8869). (**e–f**) Quantification of synapses (**e**, *P = 0.0400, **P = 0.0063) and myelinated axons (**f**, *P* = 0.5634, *P* = 0.9541) in the dLGN. (**g**) Representative images of EM in the dLGN (synapses indicated by white asterisks). Scale bars = 1 µm. Statistical differences were determined by one-way ANOVA with Holm-Šidák post-test. All data are expressed as means ± SEM including *n* = 5–6 mice per group, 3–6 fields from 2–3 sections (NeuN), and 4 fields (EM) per mouse. Bis = bisbenzimide

To explore whether there is loss of neuronal cell bodies in these thalamic regions receiving input from damaged white matter tracts during EAE, NeuN^+^ cells colocalized with Bis were quantified in the VPL (Fig. 3a) and dLGN (Fig. 3b). The number of NeuN^+^ cells was not significantly different at any time point during EAE compared to naïve controls in either the VPL (Fig. 3c) or dLGN (Fig. 3d). Vesicular glutamate transporter 2 (VGLUT2), which is enriched in the dLGN, was used in combination with structural landmarks to identify the VPL and dLGN. Quantification of age-matched naïve controls for 15, 35, and 60 dpi did not exhibit age-related differences in T cell infiltration for the VPL (Additional file 1: Figure S8a) or dLGN (Additional file 1: Figure S8b). NeuN^+^ cells in the VPL (Additional file 1: Figure S8c) or dLGN (Additional file 1: Figure S8d) were also not significantly different between age-matched naïve groups, with the exception of controls for 35 dpi compared to 60 dpi in the VPL, likely driven by one mouse in the 35 dpi group. Overall, these data provide evidence that the increase in reactive gliosis observed in the VPL and dLGN is not coincident with neuronal cell body loss, in contrast to the SC ventral horn and retina.

Although loss of neurons was not observed in the dLGN, loss of presynaptic terminals quantified by immunofluorescent staining with VGLUT2 has been previously reported in the dLGN during early EAE [33]. To further explore the timing of synaptic loss in relationship to optic nerve pathology, ultrastructural analysis of the dLGN was performed using electron microscopy. At 15 dpi there was a significant loss of synapses in the dLGN (Fig. 3e, synapses indicated by white asterisks in Fig. 3g), consistent with the timing of reactive gliosis (Additional file 1: Figure S7h,l). As optic nerve axons provide synaptic input into the dLGN, myelinated axons were counted in electron micrographs. No significant change in the number of myelinated axons was observed in the dLGN at either 15 or 35 dpi (Fig. 3f). Since there is no overt T cell infiltration nor loss of neuronal cell bodies or myelinated axons within the dLGN, these data suggest that the observed reactive gliosis in this region at 15 dpi may be in response to the adaptive inflammatory insult driving axonal loss in the optic nerve (Figs. 1 and S1d), in turn resulting in loss of axon terminals in the dLGN.

### VEP and OCT visual assessments reflect the timing of white matter and gray matter injury during EAE

**Fig. 4:**
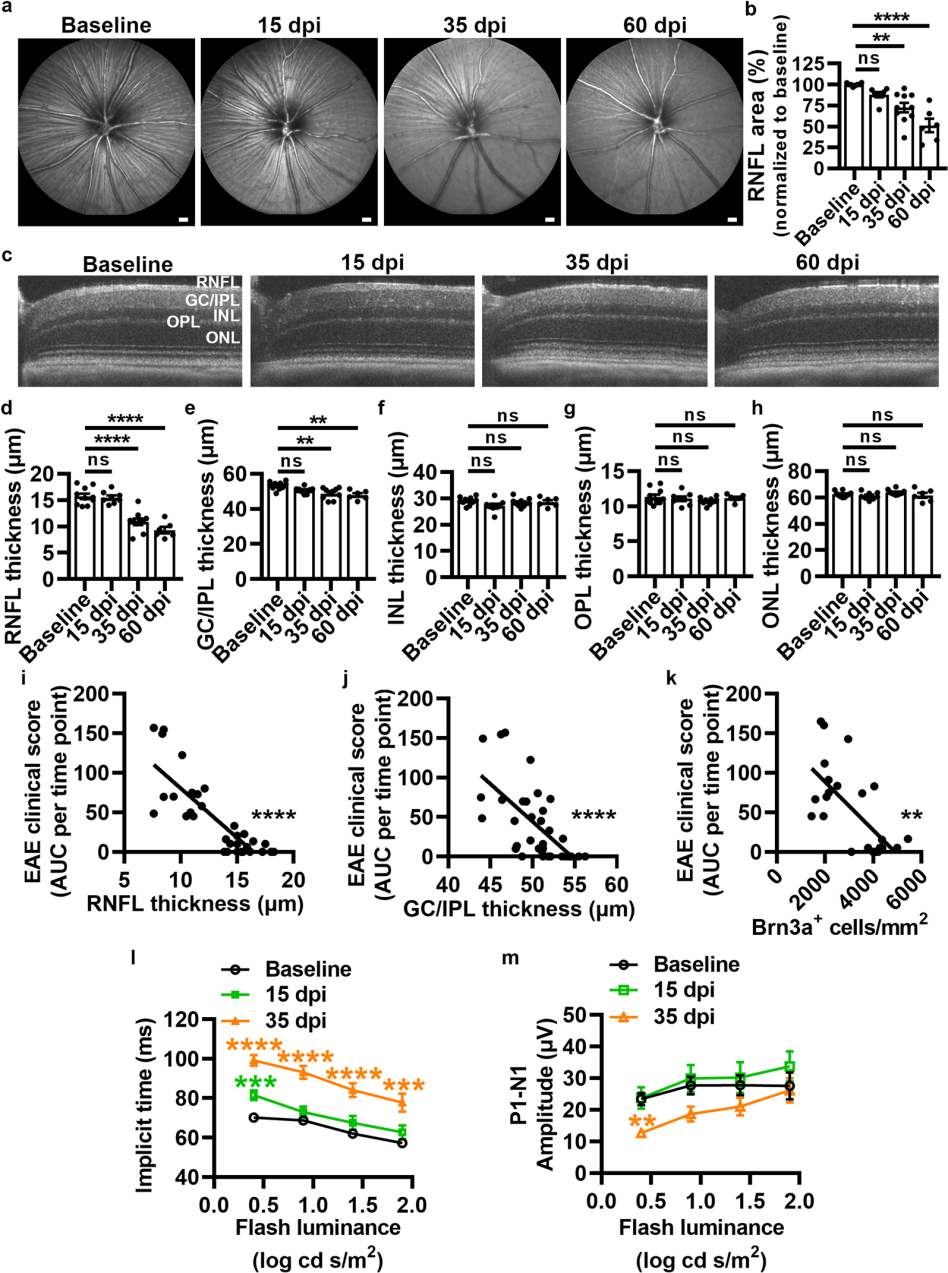
Retinal nerve fiber and ganglion cell/inner plexiform layer thicknesses are decreased during chronic EAE, while VEPs are delayed during acute and chronic EAE. **a** Representative SLO images. Scale bars = 200 µm. *P* values listed consecutively are baseline vs 15, 35, or 60 dpi, respectively **b** Quantification of RNFL area (*P* = 0.1020, **P = 0.0012, ****P < 0.0001). **c** Representative OCT b scans. **d–h** Quantification of the thicknesses of the RNFL (*P* = 0.7494, ****P < 0.0001, ****P < 0.0001), GC/IPL (*P* = 0.1083, **P = 0.0017, **P = 0.0014), INL (*P* = 0.1795, *P* = 0.8098, *P* = 0.8662), OPL (*P* = 0.8955, *P* = 0.5683, *P* = 0.9095), and ONL (*P* = 0.7659, *P* = 0.7080, *P* = 0.8720). **i–k** Correlation analyses between EAE clinical score (area under curve for each group) and RNFL thickness (**i**, Spearman r = − 0.7922, ****P < 0.0001), EAE clinical score versus GC/IPL thickness (**j**, Spearman r = − 0.6676, ****P < 0.0001), and EAE clinical score versus Brn3a^+^ cells in the retina (**k**, Spearman r = − 0.6070, **P = 0.0013) across all time points. **l–m** VEP latency (implicit time) and amplitude. *P* values for VEP data are listed in Table S1. Statistical differences were determined using a one-way ANOVA with Holm-Šidák post-hoc test (at each flash luminance for VEP) or Spearman r test for correlation analyses. All data are expressed as means ± SEM including n = 6–10 mice each for baseline and 15, 35, and 60 dpi

To explore if the pathological alterations in the visual pathway during EAE resulted in structural and functional visual deficits in EAE, visual assessments were performed at baseline and following EAE induction. The RNFL, which consists of unmyelinated axons projecting from RGCs, was evaluated as hyper-reflective fibers in 2D scanning laser ophthalmoscopy (SLO) images (Fig. 4a). The area of the fundus filled by RNFL fibers was measured, and a statistically significant reduction in RNFL area was observed beginning at 35 dpi and sustained at 60 dpi (Fig. 4b). Retinal thickness measurements from OCT images confirmed a significant decrease in the RNFL at 35 dpi (Fig. 4c,d). Additionally, OCT imaging revealed a significant reduction in the thickness of the combined GCL and IPL layers (GC/IPL, Fig. 4e) at 35 dpi consistent with histopathological assessment of RGC loss (Fig. 2h). Loss of myelinated axons in the optic nerve occurred at 15 dpi (Fig. 1o), whereas loss of RGCs occurred at 35 dpi (Fig. 2h). This is consistent with the timing of decreased RNFL area suggesting a retrograde degeneration of the unmyelinated portion of RGC axons and their cell bodies subsequent to optic nerve damage. Furthermore, there is a significant linear relationship between EAE clinical scores (EAE area under the curve measured for each group) and RNFL (Fig. 4i) and GC/IPL (Fig. 4j) thickness as well as the number of Brn3a^+^ cells (Fig. 4k), consistent with previously reported data [20, 21]. Moreover, our data demonstrate that Brn3a^+^ cell loss correlated with loss of SC neurons (Additional file 1: Figure S6a) suggesting that neurodegeneration occurs in both the visual pathway and in the SC, and that the use of OCT provides a more easily accessible, noninvasive technique to monitor neurodegeneration. In comparison, there was no change in the thicknesses of the INL (Fig. 4f), OPL (Fig. 4g), or ONL (Fig. 4h) at any EAE time point compared to baseline. VEPs evoked by light stimulation were delayed beginning at 15 dpi for the lowest flash luminance (Fig. 4l, P values listed in Table S1), coinciding with the timing of demyelination and axonal loss in the optic nerve and synaptic loss in the dLGN. At 35 dpi, delays in VEP were more prominent and significant across all stimulus conditions (Fig. 4l) and VEP amplitude was decreased at the lowest flash luminance (Fig. 4m, P values listed in Table S1), consistent with the timing of RGC loss. Taken together, these data suggest that VEP recordings detected functional deficits that aligned with loss of myelinated axons in the optic nerve during peak EAE while OCT imaging tracked retinal thinning consistent with loss of RGCs and their unmyelinated axons within the RNFL during chronic EAE. These data indicate that OCT imaging may provide a non-invasive and sensitive approach to track neurodegeneration in MS.

### OCT measures correlate with clinical disability and thalamic subregional volume in relapsing MS

OCT measures in 443 subjects with MS without a history of optic neuritis (Table 1) followed at the Mellen Center for MS were available with longitudinal neuro performance measures (median follow-up 1.2 years [IQR 2.08]). Linear mixed-effects models with a random intercept adjusting for age, sex, race, and disease duration were performed to correlate initial OCT measures with changes in neuroperformance measures (e.g. patient determined disease steps (PDDS), manual dexterity time in the dominant and non-dominant hands, and walking speed time). Including all subjects with MS, non-dominant hand manual dexterity was the measure with the greatest significant change, which correlated with initial macular volume (estimate − 2.21, 95% CI − 3.38 to − 1.02). For every 1 mm^3^ increase in macular volume, non-dominant hand manual dexterity improved by 2.2 s. This effect was similar in subjects with relapsing–remitting MS (RRMS, estimate − 2.91, 95% CI − 4.15 to − 1.66), but not significant in progressive MS. Sex was not a significant predictor in these longitudinal analyses.

**Table 1:** Characteristics of patients without history of optic neuritis for longitudinal analysis

	Overall (N = 443)
*Sex*	
Female	322 (72.7%)
Male	121 (27.3%)
*Age*	
Mean (SD)	42.2 (10.7)
Age diagnosed	
Mean (SD)	33.8 (10.4)
*Race*	
White	388 (87.6%)
Black	34 (7.7%)
Other	8 (1.8%)
Unknown	13 (2.9%)
*BMI*	
Mean (SD)	29.9 (7.7)
*MS Years*	
Mean (SD)	9.9 (8.9)
*MS Type*	
Progressive	110 (24.8%)
Relapsing	263 (59.4%)
Unknown	70 (15.8%)

As subjects with RRMS predominantly contributed to the relationship between OCT and longitudinal change with respect to neuroperformance measures, we next evaluated Spearman correlation coefficients between thalamic subregions of interest with (1) all OCT measures and (2) all neuroperformance measures in a cross-sectional analysis of only subjects with RRMS. The lateral geniculate subregion, receiving input from the optic tract, expectedly correlated with all OCT measures except for the outer retinal average thickness (Table 2). OCT measures modestly correlated with thalamic ventral posterior subregional volume, which receives ascending SC input via the spinothalamic tract (Table 3). Neuroperformance measures were weakly correlated with lateral geniculate (Table 2) and ventroposterior (Table 3) thalamic subregions as well as OCT measures (Table S2). The strongest correlation between a neuroperformance measure and thalamic subregion was also with non-dominant hand dexterity time.

**Table 2:** Correlations of the lateral geniculate (LGN) subregion with OCT and neuroperformance measures in relapsing MS

Correlation with lateral geniculate volume	n	Spearman rho coefficient	*P*	Median days	IQR days
Baseline OCT measures					
Average Ganglion Cell-Inner Plexiform (GC/IPL)	62	0.64	< 0.0001	29.0	165.5
Peripapillary RNFL	67	0.66	< 0.0001	29.0	170.5
Macular RNFL	31	0.55	0.001	21.0	83.0
Macular volume	63	0.54	< 0.0001	29.0	152.0
Outer retinal average thickness (macular scan)	31	0.03	0.85	21.0	83.0
Baseline Neuroperformance measures					
Patient determined disease steps	747	− 0.22	< 0.0001	7.0	36.0
Dominant hand manual dexterity test	662	− 0.27	< 0.0001	5.0	33.0
Nondominant hand manual dexterity test	662	− 0.28	< 0.0001	5.0	33.0
Walking speed test	651	− 0.24	< 0.0001	5.0	33.0

**Table 3:** Correlations of the ventral posterior (VP) subregion with OCT and neuroperformance measures in relapsing MS

Correlation with ventral posterior volume	n	Spearman rho coefficient	*P*	Median days	IQR days
Baseline OCT measures					
Average Ganglion Cell-Inner Plexiform (GC/IPL)	62	0.43	0.0006	29.0	165.5
Peripapillary RNFL	67	0.44	0.0002	29.0	170.5
Macular RNFL	31	0.46	0.01	21.0	83.0
Macular volume	63	0.38	0.002	29.0	152.0
Outer retinal average thickness (macular scan)	31	0.002	0.99	21.0	83.0
Baseline Neuroperformance measures					
Patient determined disease steps	747	− 0.24	< 0.0001	7.0	36.0
Dominant hand manual dexterity test	662	− 0.30	< 0.0001	5.0	33.0
Nondominant hand manual dexterity test	662	− 0.33	< 0.0001	5.0	33.0
Walking speed test	651	− 0.28	< 0.0001	5.0	33.0

Cross-sectional analysis using linear multivariable models including age, sex and disease duration were performed in subjects with relapsing MS to evaluate the association between all OCT measures (as predictors) with each neuroperformance measure (outcome). Here, macular volume correlated best with non-dominant hand manual dexterity time (estimate − 4.80, *P* = 0.04) followed by walking speed (estimate − 3.13, *P* = 0.0004), and PDDS (estimate − 1.18, *P* = 0.001); sex was not a significant correlate. Linear models correlating all thalamic subregions (predictors) with individual OCT measures (outcome) were calculated after adjusting for age, sex, and disease duration. Male sex in these models correlated with lower peripapillary RNFL (estimate − 9.16, *P* = 0.02) and higher outer retinal (estimate 1.88, *P* = 0.01) thickness. The lateral geniculate subregion correlated with GC/IPL (estimate = 0.09; *P* = 0.002), peripapillary RNFL (estimate = 0.14; *P* = 0.0002), and macular volume (estimate = 0.004; *P* = 0.03). The lateral dorsal subregion correlated with macular RNFL (estimate = 0.46; *P* = 0.02) and outer retinal thickness (estimate = − 0.44; *P* = 0.04).

We also performed linear models to evaluate the effect of global measures with T2-lesion volume (T2LV), whole brain fraction (WBF), SC area, white and gray matter fractions (WMF, GMF), and total thalamic fraction as predictors of each OCT measure (outcome) adjusting for age, sex, and disease duration in patients with a relapsing course. The only significant predictors were GC/IPL—WMF (estimate 326.4, *P* = 0.03); peripapillary RNFL—upper SC area (estimate 0.30, *P* = 0.03); macular volume—SC area (estimate 0.01, *P* < 0.05).

Overall, macular volume correlated with non-dominant hand manual dexterity time in both longitudinal and cross-sectional analyses in subjects with RRMS. OCT measures correlated as expected with the lateral geniculate subregion but also the lateral dorsal. These findings support neurodegenerative changes occurring in visual (retinal and macular measures) and thalamic subregions coinciding with clinical disability worsening.

## Discussion

Neurodegenerative changes in MS occurring throughout the disease course leads to insidious clinical disability worsening as the sequelae of inflammatory demyelinating lesions affecting tracts throughout the CNS. While prior studies have correlated visual assessments to Expanded Disability Status Scale (EDSS) and whole brain atrophy [13, 18, 34], we sought to detect potentially earlier and more subtle neurodegenerative changes affecting the visual pathway and thalamic subregions. We found that OCT measures of the inner retinal layers (RNFL and GC/IPL) correlated with thalamic subregions associated with afferent projections from the optic nerve and spinothalamic tract in patients with MS.

Several studies in patients with MS have demonstrated an association between RNFL thinning and decreased macular volume, and although this phenomenon has a stronger association in patients with a history of optic neuritis, it is also evident in patients without a history of optic neuritis [18, 35, 36]. This holds true for cerebral white matter lesions in MS, where presence of a lesion may not result in an overt clinical outcome prompting development of predictive models such as the “rule of five”, correlating five or more lesions with a relapse [37]. Furthermore, more subtle visual changes have been documented in MS patients without a history of optic neuritis to support the presence of visual pathology such as delayed VEPs and low contrast sensitivity, as well as inflammation and atrophy in postmortem optic nerves [38]. Clinical disease progression likely reflects neurodegenerative changes (loss of axons, synapses, and neurons) as the sequelae of focal lesions. Therefore, it is necessary to measure/follow neurodegeneration as current imaging biomarkers are focused on capturing inflammatory lesions. In this regard, the anatomical segregation of neuronal cell bodies in the retina from the optic nerve, which solely houses axons of RGCs that supply presynaptic input to the lateral geniculate nucleus of the thalamus, provides a unique landscape to study how white matter damage contributes to neurodegeneration.

While the murine EAE model does not have significant brain lesions, it causes demyelinating SC lesions with some animals developing hind-limb paralysis. The spinal cord is disproportionately affected in patients with progressive MS [39] and those with lateral cord lesions [40] and focal atrophy, termed “critical” [41], are due to axonal loss within the corticospinal tract leading to paraparesis. Our results show comparable T cell infiltration, demyelination, and axonal loss in SC white matter and optic nerve during peak of EAE (15 dpi), which was accompanied by a functional delay in VEPs. At this same time point, RGC loss or reactive gliosis within the inner retina (RNFL/GCL, IPL and INL) or outer retina (OPL and ONL) was not observed. However, in the chronic phase of disease (35 dpi), RGC loss and reactive gliosis were consistent with OCT imaging showing thinning of the RNFL and GC/IPL, but no change in the INL or the outer layers of the retina. Taken together, VEP delays coincided with acute loss of myelinated axons in the optic nerve while OCT imaging tracked retinal thinning and was consistent with loss of RGCs during chronic disease. Infiltration of T cells was detected in the optic nerve at peak of disease (15 dpi), but not found in the retina at any of the time points studied. Similar results have been reported in the optic nerve crush model, where loss of RGCs occurs after selective transection of the optic nerve [42]. To further support this concept, loss of PSD95, a marker of postsynaptic terminals, but not synaptophysin, a marker of presynaptic terminals was documented in the IPL during EAE [21]. Amacrine and bipolar cells provide presynaptic input to postsynaptic terminals of RGCs supporting loss of synapses associated with RGCs in the IPL. These data suggest a retrograde degeneration of RGCs following damage to the optic nerve during autoimmune demyelination. Similarly in the SC, loss of neurons in the ventral horn was not evident until the chronic phase of EAE (35 dpi) subsequent to myelin and axonal injury at peak of disease (15 dpi) in the SC white matter and consistent with retrograde loss of neuronal cell bodies detected in the visual pathway. However, neuronal cell bodies in the dLGN and VPL that receive synaptic input from axons traveling via the optic nerve and SC white matter were not lost during the peak, chronic, or sustained chronic phase of EAE. Interestingly, synaptic loss quantified by electron microscopy occurred in the dLGN consistent with microgliosis at peak of disease and coincident with myelin and axonal loss in the optic nerve. Another study reported loss of presynaptic, but not postsynaptic terminals in the dLGN during EAE [33]. These data, in conjunction with our data showing no change in neuron number and relative lack of T cell infiltration in the dLGN suggest that axonal injury drives acute loss of its axon terminals. These data are also consistent with human postmortem data showing that reduced thalamic volume is a consequence of loss of axonal input to the thalamus from lesions not directly in the thalamus [7]. While it is known that demyelination specifically in the dLGN induced by cuprizone exhibits synaptic loss [43], a reduction in the number of myelinated axons in the dLGN was not observed in EAE. This may be due to the early time points evaluated (15 and 35 dpi) in this study or the chronic nature of this model and warrants further exploration in progressive models of autoimmune demyelination or in models exhibiting forebrain pathology such as the SJL model. Nonetheless, our analysis of the EAE model suggests that synaptic loss in distal axon terminals in the dLGN occurs acutely with axonal injury in the optic nerve.

While a decrease in the thickness of the inner layers of the retina including the RNFL and the GC/IPL was observed with OCT during chronic EAE, there was no change in INL thickness, which contains cell bodies of amacrine and bipolar cells that provide presynaptic input to RGCs. This may be due to the early time points observed in these studies [20]. While the IPL cannot be differentiated from the GCL using OCT, future studies that compare the INL with the RNFL and GC/IPL could be of potential interest in monitoring neurodegeneration in MS. Evaluating the timing of changes in the INL versus the GC/IPL and RNFL over a longitudinal course in the same patient may provide insight into the progression of neurodegeneration in MS.

In patients with MS, we found non-dominant hand measures were influenced by baseline macular volume. We speculate that non-dominant hand manual dexterity may be manipulated by complementary pathways including the visual and proprioceptive systems while the dominant hand may have more robust canonical input from the corticospinal tract without a dependency on other inputs. While this effect was only observed in patients with RRMS, these data are consistent with the observation that RRMS patients who developed progressive disease showed faster cervical SC atrophy rates at least four years before conversion to progressive disease compared to those that did not progress [44]. This emphasizes the need to monitor neurodegeneration early in the RRMS disease course as a driver of clinical disability. Some limitations include a relatively small number of subjects with available OCT data and limited longitudinal follow-up which can be addressed with wider adaptation of OCT and multi-center studies. While we found significant differences predominantly in the RRMS population compared to progressive, future studies powered to separate differences between secondary and primary progressive subjects may be useful. Sex differences may account for differences in neurodegeneration as males have been shown to experience more extensive gray matter atrophy (e.g. thalamic) with an effect on manual dexterity [45, 46]. We evaluated the contribution of sex when using thalamic subregional volumes to predicting OCT outcomes and found male sex had lower peripapillary RNFL but higher outer retinal thickness. This warrants further investigation in MS and in animal models to understand if the visual system can also reflect sex-specific changes in neurodegeneration. Our results suggest more global changes in regions such as the SC and white matter fraction (WMF) reflect changes in OCT measures: upper cervical cord cross sectional area was a significant predictor of GC/IPL and peripapillary RNFL thickness and WMF was a significant predictor of GC/IPL thickness.

Measuring clinical disability as an outcome of progression underestimates neurodegenerative changes occurring independent of injury on eloquent (clinically apparent) pathways (directly or due to secondary neuro/axonal degeneration). The retina provides a timely and noninvasive window into appreciating neurodegenerative changes not otherwise perceptible. Its laminar organization also makes it amenable to monitor longitudinal structural and functional changes in concert with MRI and neuroperformance measures throughout the disease course. Elucidating the spatio-temporal paradigm of neurodegeneration will facilitate discovery of mechanisms driving this process early in the disease course necessary for therapeutic intervention. Likewise, as remyelinating and neuroprotective strategies develop, a multimodal approach to detecting their effect throughout the CNS will be necessary.

## Materials and methods

### Animals

Male mice on the C57BL/6J background were purchased from the Jackson Laboratory (Bar Harbor, ME) and used for all experiments. EAE was induced in 8- to 10-week-old mice, and age-matched naïve mice were used as untreated controls. Animals were housed and treated in accordance with the National Institutes of Health (NIH) and the Institutional Animal Care and Use Committee (IACUC) guidelines of the Cleveland Clinic.

### Induction and scoring of EAE

EAE was induced in 8- to 10-week-old male mice as previously described [47, 48] by subcutaneous injection of 50 µg MOG_35-55_ peptide emulsified in complete Freund’s adjuvant containing 125 µg desiccated *Mycobacterium tuberculosis* (Hooke Laboratories, Lawrence, MA). On days 0 and 2 of EAE, mice were injected intraperitoneally with 200 ng *Bordetella pertussis* toxin (Hooke Laboratories, Lawrence, MA) in 500µL phosphate-buffered saline (PBS). EAE symptoms were monitored daily and mice were assigned clinical scores as follows: 0 = healthy, no symptoms, 1 = loss of tail tone, 2 = flaccid tail, 3 = partial hind limb paralysis, 4 = complete hind limb paralysis, 5 = moribund animal (humanely euthanized), 6 = death. Subsets of mice were sacrificed at each time point for immunohistochemistry and electron microscopy.

### Optical coherence tomography and scanning laser ophthalmoscopy

Optical coherence tomography (OCT) was performed using an ultra-high resolution spectral domain OCT system (Envisu R2210 UHR Leica Microsystems Inc.) for in vivo cross-sectional imaging in mice. Prior to imaging mice were anesthetized by intraperitoneal injection with either pentobarbital (75 mg/kg) or a cocktail of ketamine (80 mg/kg ketamine HCL, Zoetis) and xylazine (16 mg/kg, AnaSed Injection). The corneal surface was anesthetized with 1% proparacaine HCL, and mice were placed on a temperature-regulated heating pad. Pupils were dilated with 0.5% topical tropicamide/phenylephrine combination eyedrops (Santen Pharmaceuticals, Osaka, Japan). The scan parameters were 1.8 mm by 1.8 mm rectangular volume scan, 1000 a-scans/200 b-scans averaged 3 times per b-scan. For scanning laser ophthalmoscopy (SLO), 50 µL sodium fluorescein was injected intraperitoneally (1% in saline, AL-FLUOR 10%, Akron Inc.) and retinas were imaged using a SPECTRALIS ophthalmoscope (Heidelberg Engineering, Germany). Animals were imaged prior to induction of EAE to obtain baseline retinal thickness (for OCT) and retinal nerve fiber layer area (for SLO), and followed longitudinally at 15, 35, and/or 60 days post-EAE induction (dpi). In a second experiment, OCT and SLO were performed during chronic EAE in a subset of mice at 35 dpi and another subset at 60 dpi. Mice were euthanized following visual assessments by transcardiac perfusion with 4% paraformaldehyde (PFA). Retinal thickness was measured by caliper method using InVivoVue software. OCT measurements were performed 450 µm from the optic nerve head in both the nasal and temporal directions, and in superior and inferior regions for a total of four measurements per animal. Average thicknesses from both eyes per animal were calculated for analysis. SLO images were analyzed using ImageJ software (version 1.52p) by measuring the area filled by hyper-reflective RNFL. Average RNFL area for both eyes was calculated for analysis.

For human imaging, OCT was performed using spectral domain Cirrus HD-OCT (models 4000 or 5000; Carl Zeiss Meditec, Dublin, CA). All scans were performed without pupillary dilation by trained medical technicians at the Cleveland Clinic Mellen Center. Optic disc and macular scans were obtained using the Optic Disc Cube 200 × 200 and the Macular Cube 512 × 128 protocols, respectively. Software-extracted segmented regions or layers included for analysis include: peripapillary RNFL (pRNFL), macular RNFL, ganglion cell-inner plexiform layer thickness (GCIP), outer retina thickness (ORT), and total macular volume (TMV).

### Visual evoked potentials

Visual evoked potentials (VEP) were recorded following OCT and SLO for baseline, 15 dpi, and 35 dpi according to previously described methods [49]. Briefly, VEPs were recorded using an active electrode positioned subcutaneously along the midline of the visual cortex, with a reference needle electrode placed in the cheek. A third electrode was inserted in the tail to serve as the ground lead. Responses to achromatic strobe flash stimuli presented in a ganzfeld device (LKC Technologies, Gaithersburg, MD) were recorded under light-adapted conditions. The interstimulus interval ranged from 1.1 to 6 s, increasing with stimulus luminance from 0.4 to 1.9 log cd s/m^2^. The amplifier band-pass was set at 1–100 Hz and up to 60 successive responses were averaged to obtain single VEP waveforms. The mouse VEP is dominated by a negative component, N1 [50, 51] and the implicit time of the N1 component was measured at the negative peak. The amplitude of the VEP was measured to N1 from the preceding baseline, or positive peak (P1). Averages of VEP implicit time and amplitude for each stimulus condition was calculated per animal for analysis.

### Perfusion and preparation of mouse spinal cords, optic nerves, and retinas for immunohistochemistry

Spinal cords, optic nerves, brains, and eyes were removed after transcardiac perfusion with 4% PFA in PBS. Left eyes were post-fixed in PFA for 2 h and whole retinas were subsequently removed and prepared for immunofluorescent staining (see method below). Spinal cords, optic nerves, brains, and right eyes were fixed overnight in 4% PFA and cryoprotected in 30% sucrose. These were then embedded in Optimal Cutting Temperature freezing medium (Fisher HealthCare, Waltham, MA) and snap-frozen in 2-methylbutane on dry ice. Spinal cords were cut into 6 segments for embedding. All frozen tissue was cut into 16 µm-thick sections on a cryostat (Leica Biosystems, Buffalo Grove, IL) for immunohistochemistry.

### Preparation for electron microscopy

Mice were perfused with 4% PFA and 2% glutaraldehyde in 0.1 M sodium cacodylate buffer at pH 7.4. The lumbar regions of spinal cords and whole optic nerves were processed by the Cleveland Clinic Electron Microscopy Core. Tissue was post-fixed in 1% osmium tetroxide in water, stained with 1% uranyl acetate in maleate buffer (pH 5.1), and dehydrated with ethanol and propylene oxide before being embedded in Pure Eponate 12 resin (Ted Pella Inc., Redding, CA). Ultrathin 85-nm sections were cut with a diamond knife, stained with uranyl acetate and lead citrate, and observed with a transmission electron microscope (FEI Company, Hillsboro, OR).

### Immunohistochemistry with 3,3'-Diaminobenzidine (DAB)

Immunohistochemistry with DAB was performed to stain for myelin basic protein (MBP) as previously published [52] in spinal cords and optic nerves. Antigen retrieval was performed using 10 mM citrate buffer (pH 3.0) at 37 °C for 30 min. Sections were blocked with 5% goat serum and 0.3% Triton X-100 in PBS for one hour at room temperature (RT). Rat anti-MBP (1:500; Ab7349, Abcam, Cambridge, UK) primary antibody was diluted in blocking buffer, added to sections, and incubated overnight at 4 °C. A goat anti-rat biotinylated secondary antibody (1:200, BA-9400, Vector Laboratories, Burlingame, CA) was diluted in blocking buffer and added to sections, incubating for one hour at RT. Endogenous peroxidase activity was blocked by incubating slides in 0.3% hydrogen peroxide in methanol for 10 min at RT. Antibodies were visualized using the avidin–biotin-immunoperoxidase complex (ABC) method using the VECTASTAIN ABC Kit (PK-4000, Vector Laboratories, Burlingame, CA) and DAB Peroxidase (horseradish peroxidase) Substrate Kit (SK-4100, Vector Laboratories, Burlingame, CA). Slides were serially dehydrated and mounted in Permount (SP15, Thermo Fisher Scientific, Waltham, MA).

### Immunofluorescence

Antigen retrieval was performed for anti-NeuN immunofluorescence using 10 mM citrate buffer (pH 3.0) at 37 °C for 30 min. For all stains, sections were blocked with 5% serum corresponding to the host of the secondary antibody and 0.3% Triton X-100 in PBS for one hour at RT. Primary antibodies were diluted in blocking buffer and incubated on sections overnight at 4 °C. For anti-NeuN immunofluorescence, biotinylated secondary antibodies were diluted in blocking buffer and incubated on slides for one hour at RT. Following incubation with biotinylated secondary antibodies, slides were incubated in fluorescent-conjugated streptavidin diluted in PBS wash buffer for 30 min. For all other immunofluorescence staining, fluorescent-conjugated secondary antibodies were diluted in blocking buffer and incubated on sections for one hour at RT. Slides were mounted with Fluoromount-G (0100–01, SouthernBiotech, Birmingham, AL) containing bisbenzimide (1:1000; H3569, Invitrogen, Carlsbad, CA).

Primary antibodies used included rat anti-CD3 (1:500; 16–0032-85, Invitrogen, Carlsbad, CA), mouse anti-GFAP (1:1000; 835,301, Biolegend, San Diego, CA), rabbit anti-Iba1 (1:500; 019–19,741, Wako, Richmond, VA), mouse anti-NeuN (1:500; MAB377, Millipore, Billerica, MA), rabbit anti-NeuN (1:1000; Ab177487, Abcam, Cambridge, UK), and rabbit anti-VGLUT2 (1:200; ab216463, Abcam, Cambridge, UK). Biotinylated secondary antibodies were used at 1:200 dilution and included horse anti-mouse IgG (BA-2000, Vector laboratories, Burlingame, CA) and goat anti-rabbit IgG (BA-1000, Vector Laboratories, Burlingame, CA). Alexa Fluor 647-conjugated streptavidin was used at 1:1000 dilution (lyophilized stock diluted in 1 mg/mL PBS, S21374, Invitrogen, Carlsbad, CA). All other fluorescent-conjugated secondary antibodies were used at 1:500 or 1:1000 (all purchased from Invitrogen, Carlsbad, CA) and included Alexa Fluor 488 goat anti-rat IgG (A11006), Alexa Fluor 488 goat anti-rabbit IgG (A11034), Alexa Fluor 647 goat anti-mouse IgG (A21235), Alexa Fluor 647 goat anti-rabbit IgG (A21245), and Alexa Fluor 594 goat anti-rabbit IgG (A11037).

### Staining and preparation of retinal whole mounts

Isolated fixed retinas were permeabilized for 30 min in 0.5% Triton X-100 in PBS at RT. Retinas were then blocked in 10% goat serum, 1% bovine serum albumin, and 0.5% Triton X-100 in PBS for one hour at RT. Subsequently, retinas were incubated in goat anti-mouse F(ab) fragment (1:2000;ab6668, Abcam, Cambridge, UK) for one hour at RT. Mouse monoclonal anti-Brn3a (1:100; SC-8429, Santa Cruz Biotechnology, Dallas, TX) was diluted in blocking buffer and incubated on retinas overnight at 4 °C. Fluorescent-conjugated secondary antibody (1:500; Alexa Fluor 647, A21235, Invitrogen, Carlsbad, CA) was diluted in blocking buffer and incubated on retinas for two hours at RT. Bisbenzimide (1:100; Invitrogen H3569, Carlsbad, CA) was diluted in wash buffer and added to retinas for 20 min at RT. Four incisions were made in each retina cup to allow them to lie flat on a slide, and retinas were then mounted with Fluoromount-G (0100–01, SouthernBiotech, Birmingham, AL).

### Imaging

Images for DAB immunohistochemistry of MBP in the spinal cord and optic nerve were acquired using a Leica SCN400 slide scanner at 40 × magnification. Fluorescent images were acquired using a Nikon C2 confocal microscope system (Nikon, Melville, NY) with NIS-Elements software (Nikon, Melville, NY) at 40x (for sectioned retinas, optic nerve, and spinal cord white matter), 20x (for thalamic regions-dorsal lateral geniculate nucleus (dLGN) and ventral posterolateral nucleus (VPL), and spinal cord gray matter), or 10x (for NeuN in the thalamus and spinal cord gray matter) magnification. For retinal whole mounts, confocal z-stacks of Brn3a^+^ cells were captured in the retinal ganglion cell layer at 20 × magnification with 2.50 µm thickness and 0.5 µm step size. For qualitative images of EAE lesions in the spinal cord and optic nerve, 10 µm-thick confocal z stacks were captured at 20 × and 40x, and 3D reconstructions were generated for representative images. For electron microscopy, grids were examined on a Tecnai G2 SpiritBT transmission electron microscope (FEI Company, Hillsboro, OR) operated at 60 kV. NIS-Elements software (Nikon, Melville, NY) and Adobe Photoshop (for contrast, brightness, and color adjustments) were used to create figures and process images.

### Quantification of T cell infiltration

CD3^+^ T cells were quantified blindly using the free NIH ImageJ software (version 1.52p). T cells were identified by CD3^+^ staining around bisbenzimide^+^ nuclei in every fifth or tenth 16 µm-thick serial section in the spinal cord, optic nerve, retina, dLGN, and VPL. Average number of T cells per square millimeter per animal were calculated and used for analysis.

### Quantification of reactive gliosis

Percent area staining of GFAP and Iba1 antibodies was quantified blindly using NIS-Elements software (Nikon, Melville, NY) in every fifth or tenth 16 µm-thick serial section in the spinal cord, optic nerve, retina, dLGN, and VPL. Background subtraction was performed using the rolling ball correction (set at 5.0) or background subtraction from a region of interest (ROI) and advanced denoising for each image. Thresholds were set to the same baseline and adjusted manually as necessary for each image to assess the percent area stained. In the spinal cord, optic nerve, dLGN, and VPL, percent area was quantified per whole image area. For retinas, each retinal layer was traced using bisbenzimide to define each boundary and area fractions were measured per layer and in the total retina (sum of all layers). Average areas stained per animal were calculated and used for analysis.

### Quantification of MBP immunostaining

Spinal cord and optic nerve sections were imaged at 40 × magnification and individual sections were traced for analysis. For spinal cords, only the white matter regions in lumbar segments were traced and measured. Percent area staining of MBP antibody was quantified blindly using the NIH ImageJ free software (version 1.52p). Thresholds were manually set for each image to obtain the percent area stained with MBP. Assessments were made in every fifth or tenth 16-µm serial section. The average percent MBP-negative area fraction (demyelinated area) was then calculated per animal for analysis.

### Analysis of electron microscopy

Ultrathin 85 nm sections were imaged at × 2900 magnification in the ventral funiculus of the spinal cord, at × 11,000 in the optic nerve, and at × 18,500 in the dLGN. Axons were counted blindly in five to six images per animal for the spinal cord and optic nerve. Myelinated and unmyelinated axons were counted in each image and analyzed, which were then combined to provide a total axon count. Synapses and myelinated axons were counted blindly in four images per animal for the dLGN using the free NIH ImageJ software (version 1.52p). Average number of axons or synapses per square millimeter was calculated for each animal and used for analysis.

### Quantification of retinal ganglion cells and neuronal nuclei

RGCs and neuronal nuclei were counted blindly using the NIH ImageJ free software (version 1.52p). For retinal whole mounts, 2.50 µm-thick z stacks were compressed into a two-dimensional maximum intensity projection to visualize the RGC layer. RGCs were identified as Brn3a^+^ cells colocalized with bisbenzimide^+^ nuclei and were counted blindly in 12 fields per retina as previously described [25]. Briefly, four incisions were made in the retina to form four leaflets. Three images per leaflet were taken to represent the area at 1/6, 3/6, and 5/6 of the retinal radius from the center of each quadrant (central, medial, and peripheral positions). Total Brn3a^+^ cells per retina were used to calculate the number of RGCs per square millimeter. Neuronal nuclei were counted in the spinal cord gray matter, dLGN, and VPL from every tenth 16-µm serial section. For spinal cords, the ventral horns of the gray matter were traced and neurons were counted within the outlined region. The dLGN was traced using VGLUT2 as a marker, which is enriched in the dLGN, and neurons were counted in the entire dLGN for each section. Using anatomical landmarks and VGLUT2 staining, a single image was taken per VPL region in each serial section and neurons were counted in the whole image. Average number of NeuN^+^ neurons were calculated per animal and the number of neurons per square millimeter was used for analysis.

### Multiple sclerosis patient population

Patients with MS at the Cleveland Clinic Mellen Center for Multiple Sclerosis Treatment and Research (Cleveland, Ohio, USA) undergo tablet based neuroperformance (e.g. patient determined disease steps, manual dexterity time in the dominant and non-dominant hands, and walking speed time) as part of their routine clinical visits (25,046,650, 31,054,035). Data were obtained from visits between September 2015 and July 2021 as part of routine clinical care, therefore informed consent was not required. Approval from an ethical standards committee (Cleveland Clinic Institutional Review Board) to conduct this study was received (#18–313). Neuroperformance data were merged with OCT and MRI data where applicable for longitudinal and cross-sectional analyses.

### Magnetic resonance imaging (MRI) and image processing

MRI was acquired from patients with MS using a standard protocol on 3 T Siemens scanners and included 3D T1-weighted Magnetization Prepared RApid Gradient Echo (MPRAGE, echo time: 2.98 ms, repetition time: 2300 ms, inversion time: 900 ms, flip angle 9°, and 1mm^3^ isotropic. Data was transfer to analysis lab in the Lerner Research Institute for analysis. Thalamic subregions were segmented using MAGeT segmentation algorithm [53]. Briefly, MAGeT pipeline is a multi-atlas label fusion algorithm where the gold standard atlas was generated with a comparison to histology (https://github.com/CoBrALab) [54]. The atlas includes labels for lateral geniculate and ventral posterior subregions. MAGeT requires intermediate templates, and for this study, we randomly selected 30 intermediate images with varying degree of brain volume and measured thalamic subregional volumes from segmentation.

### Statistical analysis

Statistical analyses for EAE data were performed using GraphPad Prism software version 9.0.0. (GraphPad Software, La Jolla, CA). Specific analyses performed including *P-* values are reported where indicated. BioRender was used to generate Fig. 1a. Naïve control groups in statistical analyses correspond to the age-matched control group for the latest EAE time point included in the analysis. Clinical MS data were analyzed using R Studio 2022.02.0 + 443/R 3.0.1 (R Core Team. R: A Language and Environment for Statistical Computing. 2013. http://www.r-project.org/). Analyses included linear mixed effects and multivariable models adjusting for MS disease characteristics and demographics and Spearman correlation coefficients; details provided in results.

## Supplementary Information

**Additional file1**.**Table S1**: P values for VEP latency and amplitude during acute and chronic EAE.**Table S2**: Correlations of OCT and neuroperformance in relapsing MS.**Figure S1**: Statistical analyses show correlation between spinal cord white matter and optic nerve EAE pathology, loss of total axons, and comparable age-matched naïve control groups.**Figure S2**: Reactive gliosis occurs similarly in the spinal cord white matter and optic nerve during acute and chronic EAE.**Figure S3**; Acute and chronic lesions are similar in the spinal cord and optic nerve during EAE. **Figure S4**: Spinal cord white matter and optic nerve show inflammatory demyelinated lesions during chronic EAE.**Figure S5**: Reactive gliosis in the spinal cord gray matter and retina during EAE.**Figure S6**; Correlation between numbers of retinal ganglion cells and ventral gray matter spinal cord neurons during EAE and comparable age-matched naïve control groups. **Figure S7**: Reactive gliosis in the absence of CD3+ T cell infiltration in the dLGN during EAE.**Figure S8**: Quantification of CD3+ T cells and NeuN+ cells in age-matched naïve control groups in the dLGN and VPL.
