# Correction to: Visual imaging as a predictor of neurodegeneration in experimental autoimmune demyelination and multiple sclerosis

**DOI:** 10.1186/s40478-022-01405-9

**Published:** 2022-07-15

**Authors:** Gabrielle M. Mey, Kirsten S. Evonuk, McKenzie K. Chappell, Laura M. Wolfe, Rupesh Singh, Julia C. Batoki, Minzhong Yu, Neal S. Peachey, Bela Anand‑Apte, Robert Bermel, Daniel Ontaneda, Kunio Nakamura, Kedar R. Mahajan, Tara M. DeSilva

**Affiliations:** 1 Department of Neurosciences, Lerner Research Institute, Cleveland Clinic Foundation, and Case Western Reserve University, 9500 Euclid Avenue, Cleveland, OH 44195 USA; 2 Cole Eye Institute, Cleveland Clinic Foundation, Cleveland, OH USA; 3 Department of Ophthalmology, Cleveland Clinic Lerner College of Medicine of Case Western Reserve University, Cleveland, OH USA; 4 Louis Stokes Cleveland VA Medical Center, Cleveland, OH USA; 5 Mellen Center for MS Treatment and Research, Neurological Institute, Cleveland Clinic Foundation, Cleveland, OH USA; 6 Department of Biomedical Engineering, Lerner Research Institute, Cleveland Clinic Foundation, Cleveland, OH USA; 7 Present Address: Hooke Laboratories, Inc, Lawrence, MA USA

## Correction to: Acta Neuropathologica Communications (2022) 10:87 https://doi.org/10.1186/s40478-022-01391-y

Following publication of the original article [1], two missing sections were identified in the back matter of the article: the ‘Author contributions’ section and the ‘Competing interests’ section.

The statement in the ‘Author contributions’ section should read: GMM performed all EAE experiments and collected and analyzed IHC, IF, and EM data for all experiments, produced all figures from EAE data, and wrote manuscript draft. KSE assisted with initial EAE experiment and analyzed GFAP and Iba1 data in mouse thalamus. LMW and MKC assisted with cell counting and data analysis for CD3 and NeuN staining. RS, JCB, and BAA performed OCT imaging and analysis for rodent studies and contributed to the final revisions of the manuscript. MY and NSP recorded and analyzed VEPs from rodent studies and contributed to final revisions of the manuscript. RB, DO, and KN provided MS patient data as well as MRI imaging analysis and contributed to final manuscript revision. KRM performed analyses of human OCT, correlative analysis with MRI and neuroperformance measures, and contributed to conceptualization of research and writing of final manuscript. TMD conceptualized the research, oversaw all research, and wrote, revised, and edited the final manuscript. All authors read and approved the final manuscript.

The statement in the ‘Competing interests’ section should read: Dr. Robert Bermel discloses consultation for Astra Zeneca, Biogen, EMD Serono, Genzyme/Sanofi, Genentech/Roche, LabCorp, Novartis, TG Therapeutics, and VielaBio. Dr. Robert Bermel also discloses research support from Biogen, Genentech, and Novartis, and shares rights to intellectual property underlying the Multiple Sclerosis Performance Test, currently licensed to Qr8 Health and Biogen. Dr. Daniel Ontaneda discloses research support from Genentech, Genzyme, and Novartis and consulting fees from Biogen Idec, Genentech/Roche, Genzyme, Janssen, Novartis, and Merck. Dr. Kunio Nakamura discloses personal compensation for licensing from Biogen and research support from Genzyme and Biogen. All other authors declare no competing interests.

The original article [1] has been updated.
