# Supplementary material for: Visual imaging as a predictor of neurodegeneration in experimental autoimmune demyelination and multiple sclerosis
Source: Acta Neuropathol Commun. 2022 Jun 15;10:87. doi: 10.1186/s40478-022-01391-y (PMC9199245; doi:10.1186/s40478-022-01391-y)
Supplement: Supplementary file 1 — Additional file1.Table S1: P values for VEP latency and amplitude during acute and chronic EAE.Table S2: Correlations of OCT and neuroperformance in relapsing MS.Figure S1: Statistical analyses show correlation between spinal cord white matter and optic nerve EAE pathology, loss of total axons, and comparable age-matched naïve control groups.Figure S2: Reactive gliosis occurs similarly in the spinal cord white matter and optic nerve during acute and chronic EAE.Figure S3; Acute and chronic lesions are similar in the spinal cord and optic nerve during EAE. Figure S4: Spinal cord white matter and optic nerve show inflammatory demyelinated lesions during chronic EAE.Figure S5: Reactive gliosis in the spinal cord gray matter and retina during EAE.Figure S6; Correlation between numbers of retinal ganglion cells and ventral gray matter spinal cord neurons during EAE and comparable age-matched naïve control groups. Figure S7: Reactive gliosis in the absence of CD3+ T cell infiltration in the dLGN during EAE.Figure S8: Quantification of CD3+ T cells and NeuN+ cells in age-matched naïve control groups in the dLGN and VPL. [file 40478_2022_1391_MOESM1_ESM.pdf]

Table S1. ***P*** **values for VEP latency and amplitude during acute and chronic EAE**

	***P* value (latency)**		***P* value (amplitude)**	
**Flash Luminance ($\log cd\ s/m^2$)**	**Baseline vs 15 dpi**	**Baseline vs 35 dpi**	**Baseline vs 15 dpi**	**Baseline vs 35 dpi**
**0.4**	0.0009***	< 0.0001****	0.9156	0.0094**
**0.9**	0.2460	< 0.0001****	0.6627	0.1125
**1.4**	0.1684	< 0.0001****	0.6421	0.3596
**1.9**	0.2113	0.0004***	0.5698	0.8222

Table S2. Correlations of OCT and neuroperformance in relapsing MS

	n	Spearman rho coefficient	*P*	Median days	IQR days
**Patient determine disease steps**					
Average Ganglion Cell-Inner Plexiform (GC/IPL)	239	-0.29	**<0.0001**	16	133.5
Peripapillary RNFL	255	-0.27	**<0.0001**	18	140.5
Macular RNFL	121	-0.37	**<0.0001**	0	89.0
Macular volume	242	-0.25	**<0.0001**	15.5	142.8
Outer retinal average thickness (macular scan)	121	0.07	0.42	0	89.0
**Dominant hand manual dexterity test**					
Average Ganglion Cell-Inner Plexiform (GC/IPL)	228	-0.24	**0.0002**	16.5	133.0
Peripapillary RNFL	243	-0.19	**0.003**	18.0	133.5
Macular RNFL	117	-0.26	**0.004**	0	89.0
Macular volume	231	-0.26	**<0.0001**	16.0	135.0
Outer retinal average thickness (macular scan)	117	-0.03	0.75	0	89.0
**Non-dominant hand manual dexterity test**					
Average Ganglion Cell-Inner Plexiform (GC/IPL)	228	-0.26	**<0.0001**	16.5	133.0
Peripapillary RNFL	243	-0.15	**0.02**	18.0	133.5
Macular RNFL	117	-0.19	**0.04**	0	89.0
Macular volume	231	-0.29	**< 0.0001**	16.0	135.0
Outer retinal average thickness (macular scan)	117	-0.16	0.09	0	89.0
**Walking speed test**					
Average Ganglion Cell-Inner Plexiform (GC/IPL)	228	-0.13	0.05	16.5	130.8
Peripapillary RNFL	242	-0.10	0.12	18.0	133.0
Macular RNFL	166	-0.31	**0.0007**	0	89.5
Macular volume	231	-0.22	**0.0006**	16.0	133.5
Outer retinal average thickness (macular scan)	116	-0.19	0.05	0	89.5

Supplementary Figures

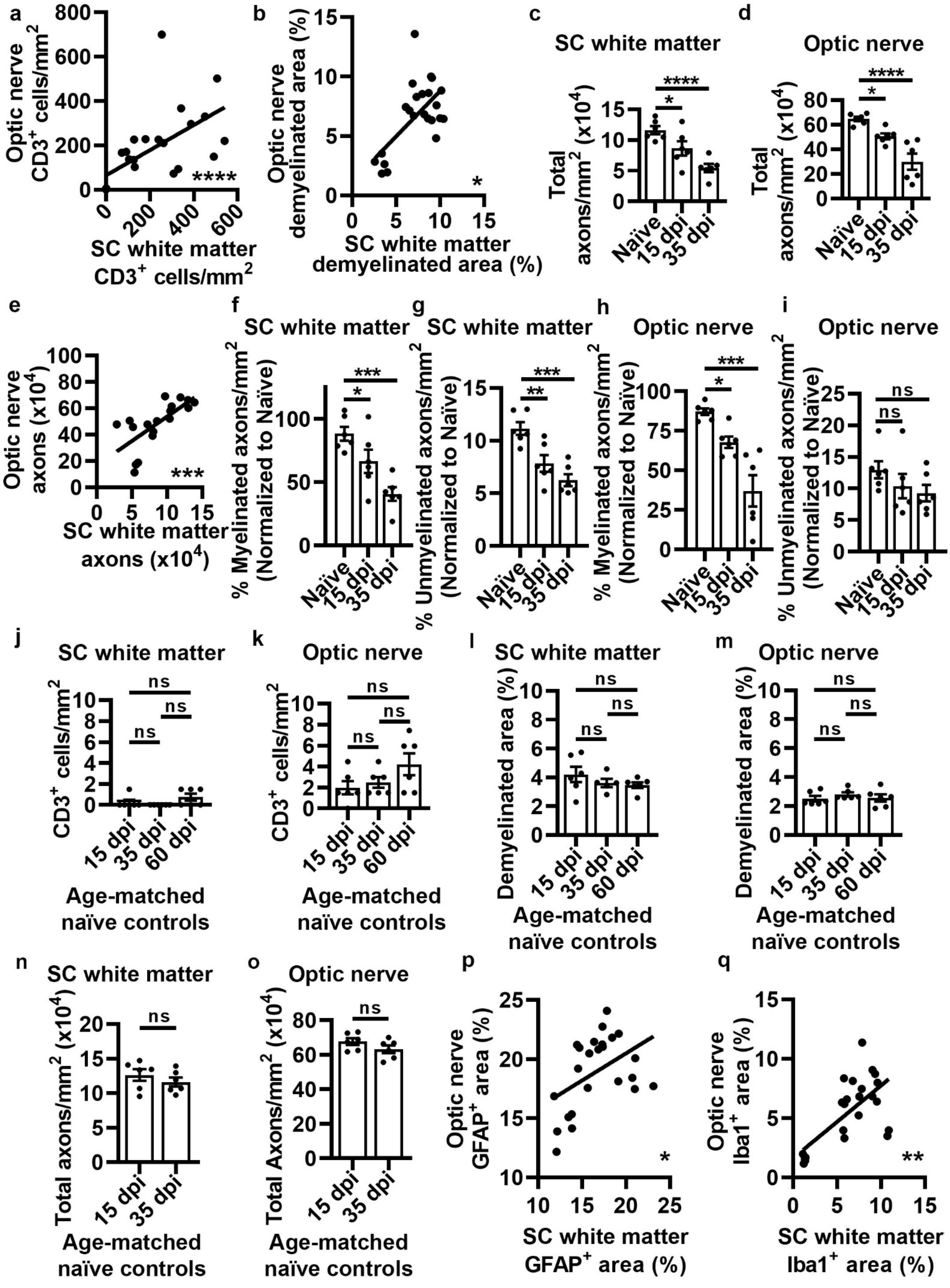

Figure S1. Statistical analyses show correlation between spinal cord white matter and optic nerve EAE pathology, loss of total axons, and comparable age-matched naïve control groups. (**a-b**) Correlation analyses comparing $CD3^+$ T cells in the SC white matter and ON (**a**, Spearman r = 0.7161, ****$P < 0.0001$) and demyelinated area in the SC white matter and ON (**b**, Spearman r = 0.4609, *$P = 0.0234$) across all time points. (**c-d**) Quantification of total axons in the SC white matter (**c**, naïve vs 15 dpi *$P = 0.0456$, naïve vs 35 dpi ****$P < 0.0001$) and ON (**d**, naïve vs 15 dpi *$P = 0.0299$, naïve vs 35 dpi ****$P < 0.0001$). (**e**) Correlation analysis comparing total axons in the SC white matter and ON (Spearman r = 0.7362, ***$P = 0.0005$). *P* values listed consecutively for age-matched naïve controls represent naïve for 15 vs 35 dpi, 15 vs 60 dpi, or 35 vs 60 dpi, respectively. (**f-i**) Percentages of myelinated and unmyelinated axons in the SC white matter and ON normalized to naïve total axons. Myelinated SC white matter (**f**) naïve vs 15 dpi *$P = 0.0425$, naïve vs 35 dpi ***$P = 0.0006$; unmyelinated SC white matter (**g**) naïve vs 15 dpi ***$P = 0.0004$, naïve vs 35 dpi **$P = 0.0023$. Myelinated ON (**h**) naïve vs 15 dpi *$P = 0.0433$, naïve vs 35 dpi ***$P = 0.0001$; unmyelinated ON (**i**) naïve vs 15 dpi $P = 0.4455$, naïve vs 35 dpi $P = 0.3026$. (**j-o**) Quantification of age-matched naïve control groups for $CD3^+$ T cells (**j**, SC: $P = 0.4751$ $P = 0.3016$, $P = 0.1272$; **k**, ON**:** $P = 0.6523$, $P = 0.1580$, $P = 0.2385$), demyelinated area (**l**, SC: $P = 0.4997$, $P = 0.4308$, $P = 0.7823$; **m**, ON: $P = 0.6811$, $P = 0.8701$, $P = 0.6811$), and total axons (**n**, SC naïve for 15 vs 35 dpi: $P = 0.3628$; **o**, ON naïve for 15 vs 35 dpi: $P = 0.1672$). (**p-q**) Correlation analysis comparing reactive gliosis by $GFAP^+$ area (**p**, Spearman r = 0.4843, *$P = 0.0165$) and $Iba1^+$ area (**q**, Spearman r = 0.6167, **$P = 0.0013$) in the SC white matter and ON. Statistical differences were determined by Spearman r test, one-way ANOVA with Holm-Šidák post-hoc test, or Student's t test. All data are expressed as means ± SEM including $n = 6$ mice per group, 6 fields (EM), 12-16 fields from 3-4 sections (CD3), and 3-4 sections (MBP demyelinated area) per mouse.

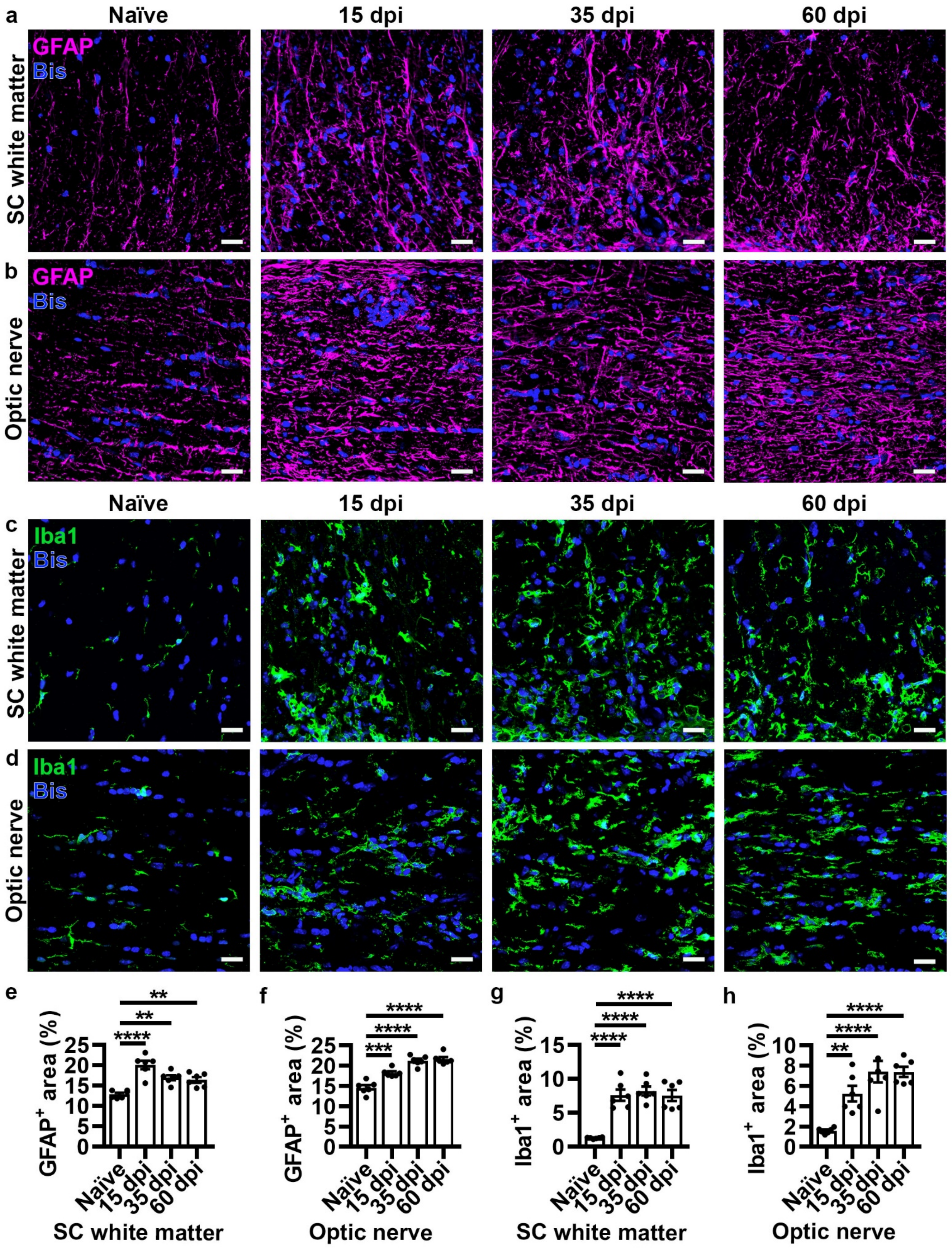

a
Naïve
15 dpi
35 dpi
60 dpi
GFAP
Bis
SC white matter
b
GFAP
Bis
Optic nerve
Naïve
15 dpi
35 dpi
60 dpi
c
Iba1
Bis
SC white matter
d
Iba1
Bis
Optic nerve
e
GFAP+ area (%)
Naïve
15 dpi
35 dpi
60 dpi
SC white matter
f
GFAP+ area (%)
Optic nerve
g
Iba1+ area (%)
SC white matter
h
Iba1+ area (%)
Optic nerve

Figure S2. Reactive gliosis occurs similarly in the spinal cord white matter and optic nerve during acute and chronic EAE. *P* values listed consecutively are naïve vs 15, 35, or 60 dpi, respectively. (**a-d**) Representative images of GFAP and Iba1 immunostaining in the SC white matter and ON. Scale bars = 20 µm. (**e-f**) Quantification of $GFAP^+$ area (**e**, SC: ****$P < 0.0001$, **$P = 0.0035$, **$P = 0.0069$; **f**, ON: ***$P = 0.0003$, ****$P < 0.0001$, ****$P < 0.0001$). (**g-h**) Quantification of $Iba1^+$ area (**g**, SC: ****$P < 0.0001$, ****$P < 0.0001$, ****$P < 0.0001$; **h**, ON: **$P = 0.0059$, ****$P < 0.0001$, ****$P < 0.0001$). Statistical differences were determined by one-way ANOVA with Holm-Šidák post-hoc test. All data are expressed as means ± SEM including $n = 6$ mice per group, 12-16 fields from 3-4 sections per mouse. Bis = bisbenzimide.

a

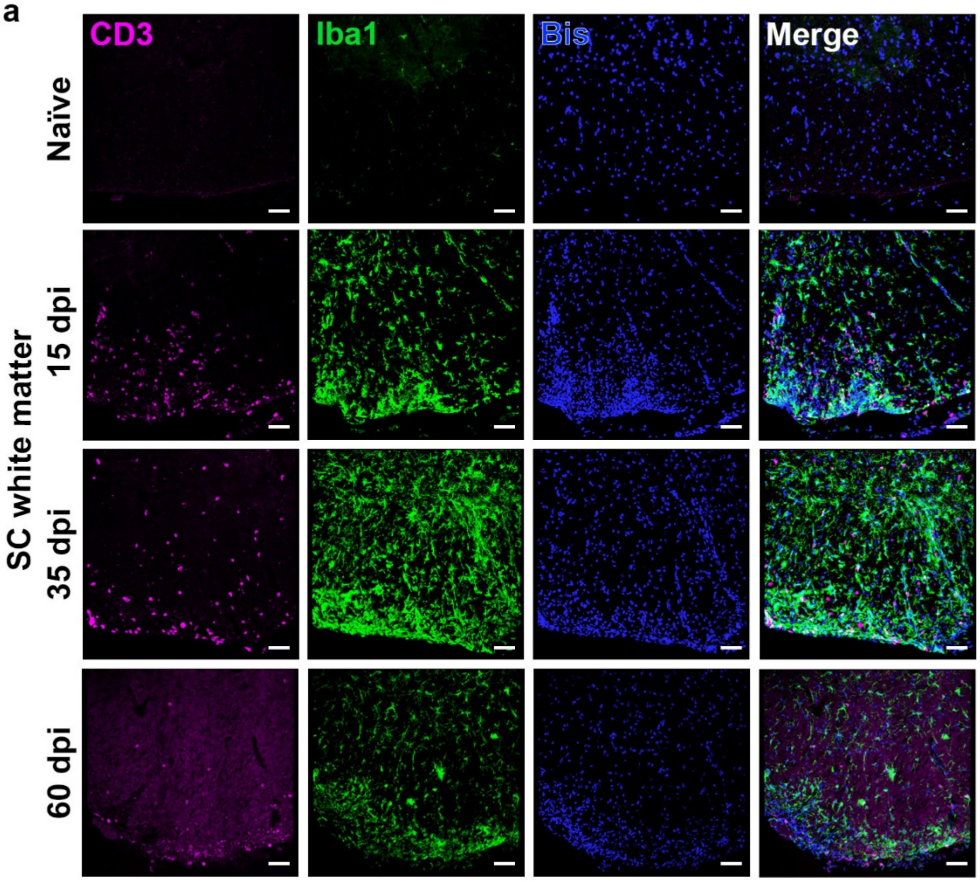
CD3
Iba1
Bis
Merge
Naïve
15 dpi
35 dpi
60 dpi
SC white matter

b

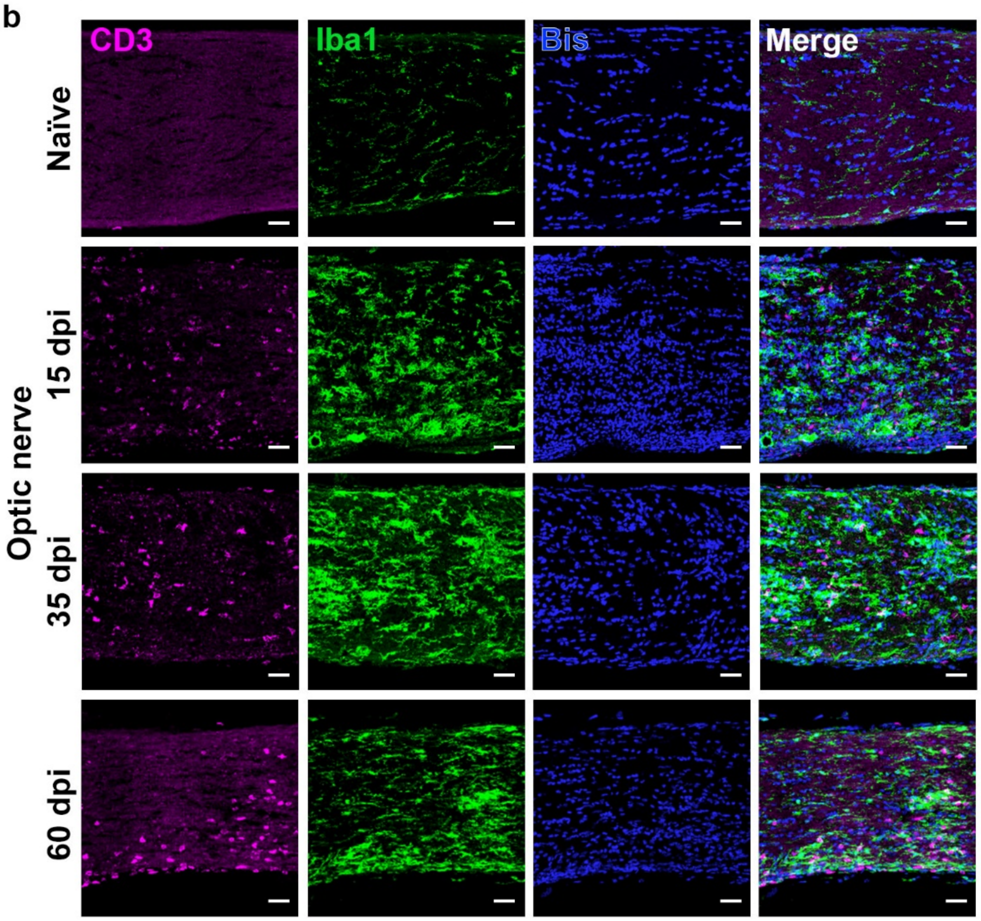
CD3
Iba1
Bis
Merge
Naïve
15 dpi
35 dpi
60 dpi
Optic nerve

Figure S3. Acute and chronic lesions are similar in the spinal cord and optic nerve during EAE. (**a**) Representative 20x images from 3D confocal reconstructions of immunofluorescence staining for $CD3^+$ T cells and $Iba1^+$ macrophages/microglia colocalized with $bisbenzimide^+$ nuclei in the SC white matter in naïve mice and at 15, 35, and 60 dpi to characterize the time course of inflammatory lesions (merge shown in far right column). This is compared to representative 20x 3D confocal images of CD3 and Iba1 immunofluorescence staining colocalized with $bisbenzimide^+$ nuclei in the optic nerves of naïve mice and at 15, 35, and 60 dpi (**b**). Dimensions of all 3D reconstructions = 511.81 µm x 511.81 µm x 10.20 µm. Scale bars = 50 µm. Bis = bisbenzimide.

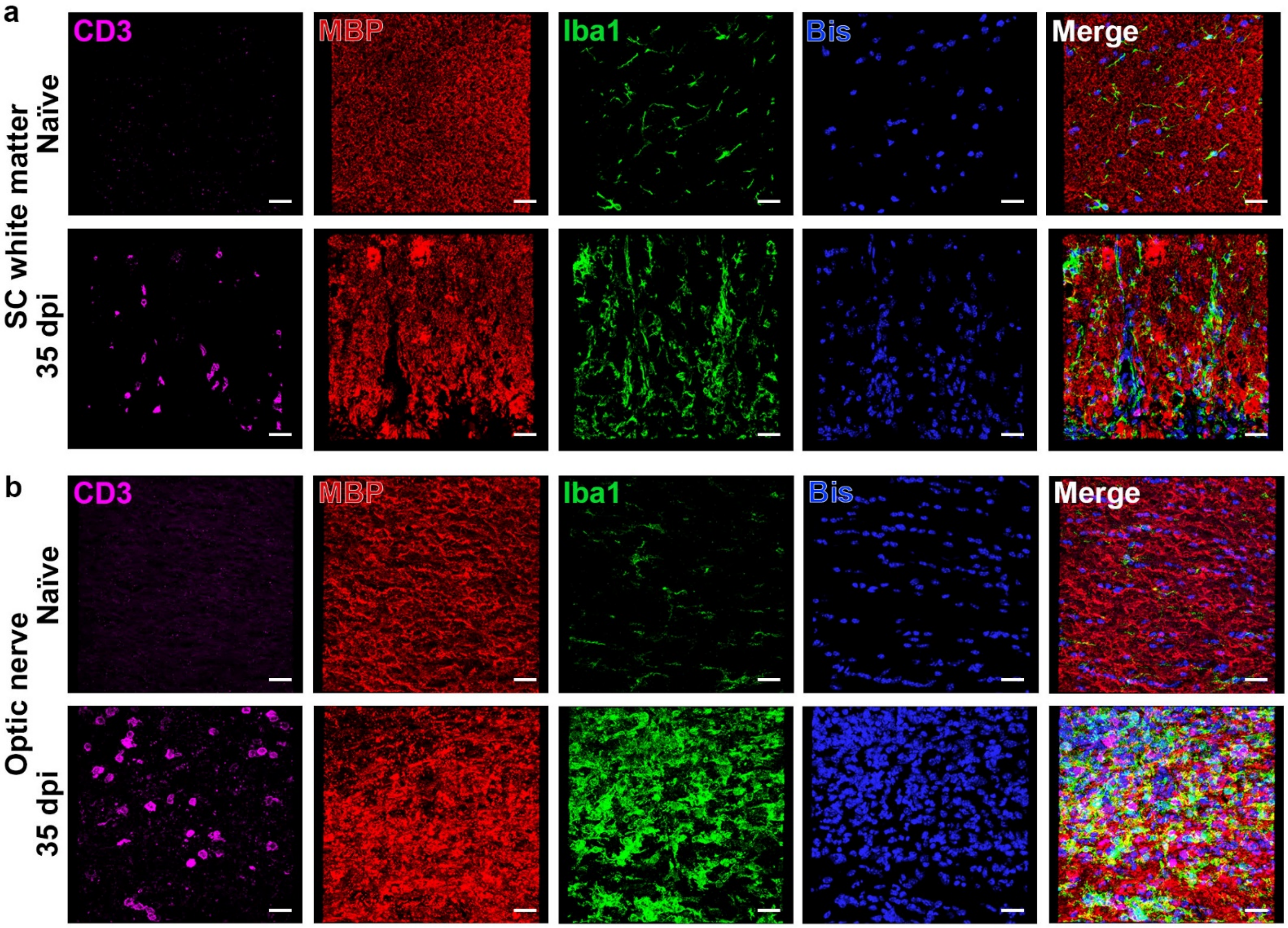

Figure S4. Spinal cord white matter and optic nerve show inflammatory demyelinated lesions during chronic EAE. (**a**) Representative 40x images from confocal 3D reconstructions of immunofluorescence staining for $CD3^+$ T cells, MBP, and $Iba1^+$ macrophages/microglia colocalized with $bisbenzimide^+$ nuclei in the SC white matter in naïve mice and at 15, 35, and 60 dpi to characterize demyelinated lesions during chronic EAE (merged in far right column). This is compared to representative 40x 3D confocal images of CD3, MBP, and Iba1 immunofluorescence staining colocalized with $bisbenzimide^+$ nuclei in the optic nerve of naïve mice and at 15, 35, and 60 dpi (**b**). Dimensions of all 3D reconstructions = 204.89 µm x 204.89 µm x 10.20 µm. Scale bars = 20 µm. Bis = bisbenzimide, MBP = myelin basic protein.

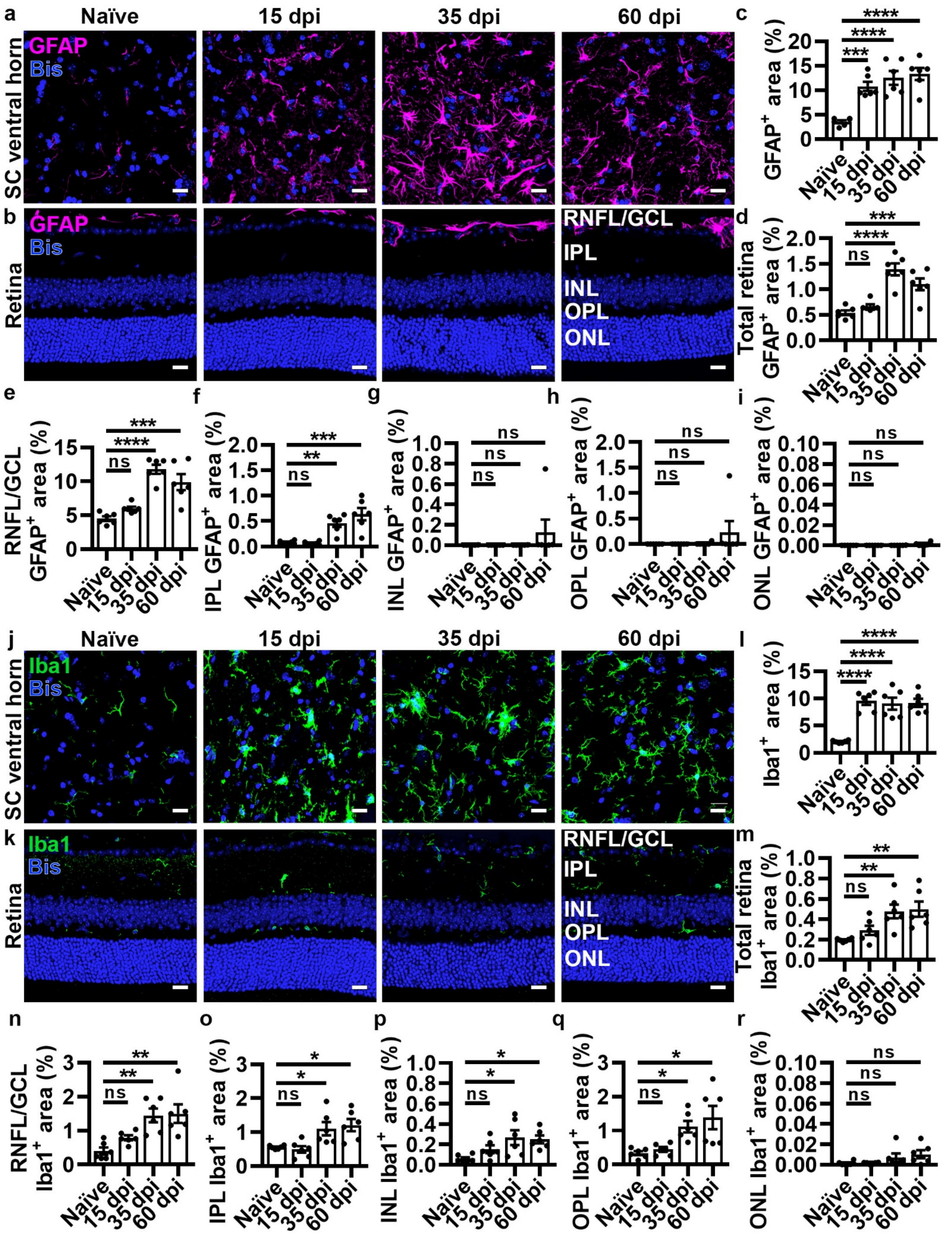

a
Naïve
15 dpi
35 dpi
60 dpi
GFAP
Bis
SC ventral horn
c
GFAP⁺ area (%)
Naïve
15 dpi
35 dpi
60 dpi
***
****
****
b
GFAP
Bis
Retina
RNFL/GCL
IPL
INL
OPL
ONL
d
Total retina GFAP⁺ area (%)
ns
****
***
e
RNFL/GCL GFAP⁺ area (%)
f
IPL GFAP⁺ area (%)
g
INL GFAP⁺ area (%)
h
OPL GFAP⁺ area (%)
i
ONL GFAP⁺ area (%)
j
Iba1
Bis
SC ventral horn
l
Iba1⁺ area (%)
k
Iba1
Bis
Retina
m
Total retina Iba1⁺ area (%)
n
RNFL/GCL Iba1⁺ area (%)
o
IPL Iba1⁺ area (%)
p
INL Iba1⁺ area (%)
q
OPL Iba1⁺ area (%)
r
ONL Iba1⁺ area (%)

Figure S5. Reactive gliosis in the spinal cord gray matter and retina during EAE. *P* values listed consecutively are naïve vs 15, 35, or 60 dpi, respectively. (**a-b**) Representative images of GFAP immunostaining in the SC ventral horn and retina with retinal layers labeled. Scale bars = 20 µm. (**c-d**) Quantification of $GFAP^+$ area (**c**, SC: ***$P = 0.0004$, ****$P < 0.0001$, ****$P < 0.0001$; **d**, total retina: $P = 0.3255$, ****$P < 0.0001$, ***$P = 0.0008$). (**e-i**) Quantification of $GFAP^+$ area in RNFL/GCL (**e**, $P = 0.1517$, ****$P < 0.0001$, ***$P = 0.0001$), IPL (**f**, $P = 0.8854$, **$P = 0.0039$, ***$P = 0.0001$), INL (**g**, $P > 0.9999$, $P > 0.9999$, $P = 0.6556$), OPL (**h**, $P = 0.9998$, $P = 0.9998$, $P = 0.6650$), and ONL (**i**, $P > 0.9999$, $P = 0.9993$, $P = 0.2135$). (**j-k**) Representative images of Iba1 immunostaining in the SC ventral horn and retina with retinal layers labeled. (**l-m**) Quantification of $Iba1^+$ area (**l**, SC: ****$P < 0.0001$, ****$P < 0.0001$, ****$P < 0.0001$; **m**, total retina: $P = 0.4314$, **$P = 0.0096$, **$P = 0.0061$). (**n-r**) Quantification of $Iba1^+$ area in RNFL/GCL (**n**, $P = 0.2617$, **$P = 0.0027$, **$P = 0.0020$), IPL (**o**, $P = 0.8412$, *$P = 0.0385$, *$P = 0.0196$), INL (**p**, $P = 0.3981$, *$P = 0.0281$, *$P = 0.0429$), OPL (**q**, $P = 0.6978$, *$P = 0.0424$, **$P = 0.0069$), and ONL (**r**, $P = 0.9595$, $P = 0.5148$, $P = 0.1896$). Statistical differences were determined by one-way ANOVA with Holm-Šidák post-hoc test. All data are expressed as means ± SEM including $n = 6$ mice per group, 12-16 fields from 3-4 sections per mouse. Bis = bisbenzimide.

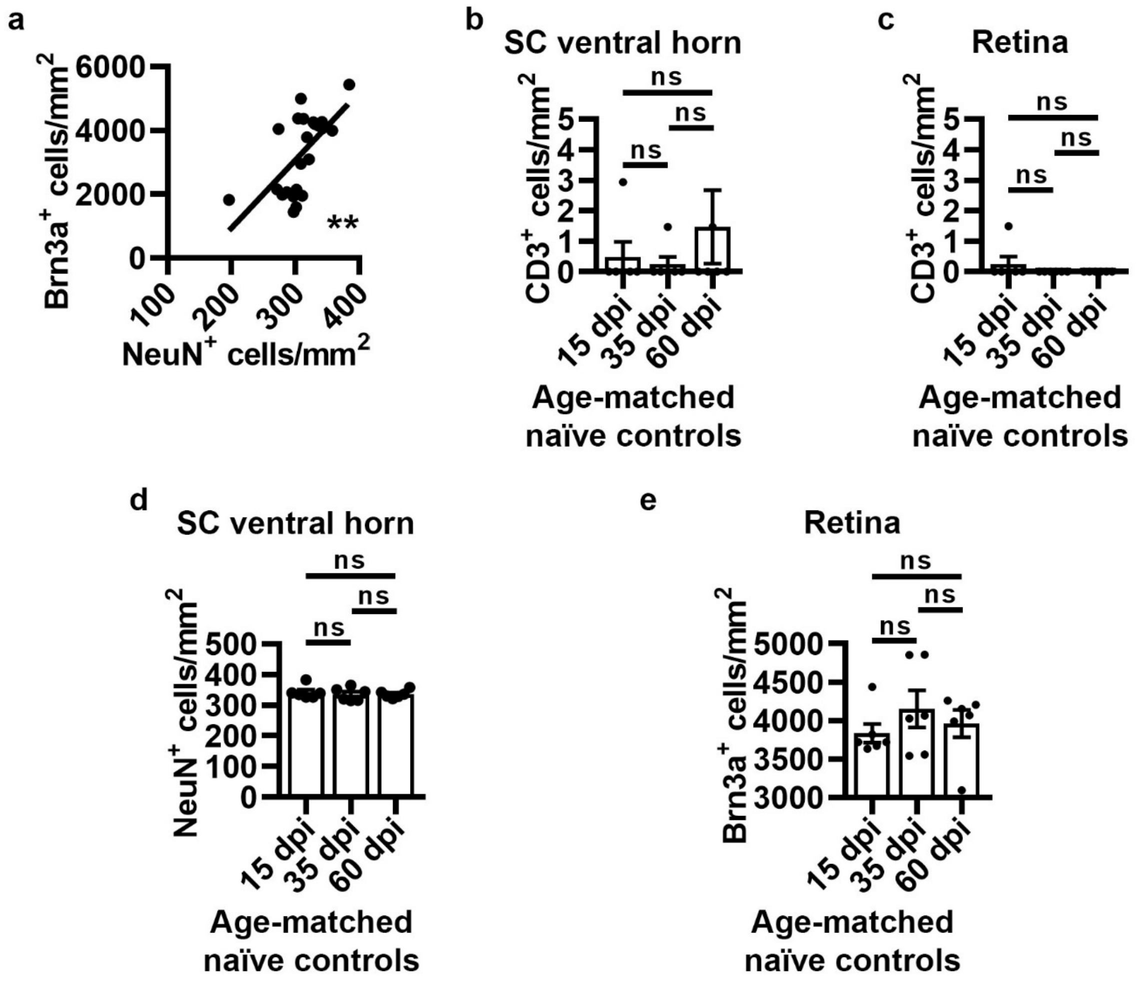

Figure S6. Correlation between numbers of retinal ganglion cells and ventral gray matter spinal cord neurons during EAE and comparable age-matched naïve control groups. (**a**) Correlation analysis comparing Brn3a$^+$ cells in the retina and NeuN$^+$ cells in the spinal cord (Spearman r = 0.6324, $^{**}P = 0.0012$). *P* values listed consecutively for age-matched naïve controls represent naïve for 15 vs 35 dpi, 15 vs 60 dpi, or 35 vs 60 dpi, respectively. (**b-e**) Quantification of CD3$^+$ T cells (**b**, SC: P = 0.8232, P = 0.6163, P = 0.6163; **c**, total retina: P = 0.5603, P = 0.5603, P > 0.9999), NeuN$^+$ cells (**d**, SC: P = 0.9108, P = 0.9108, P = 0.9485), and Brn3a$^+$ cells (**e**, retina; P = 0.5773, P = 0.7366, P = 0.7366) across age-matched naïve control groups. Statistical differences were determined by Spearman r test or one-way ANOVA with Holm-Šidák post-hoc test. All data are expressed as means ± SEM including *n* = 6 mice per group, 12-16 fields from 3-4 sections (CD3), 4-6 fields from 2-3 sections (NeuN), and 12 fields from one retina (Brn3a) per mouse.

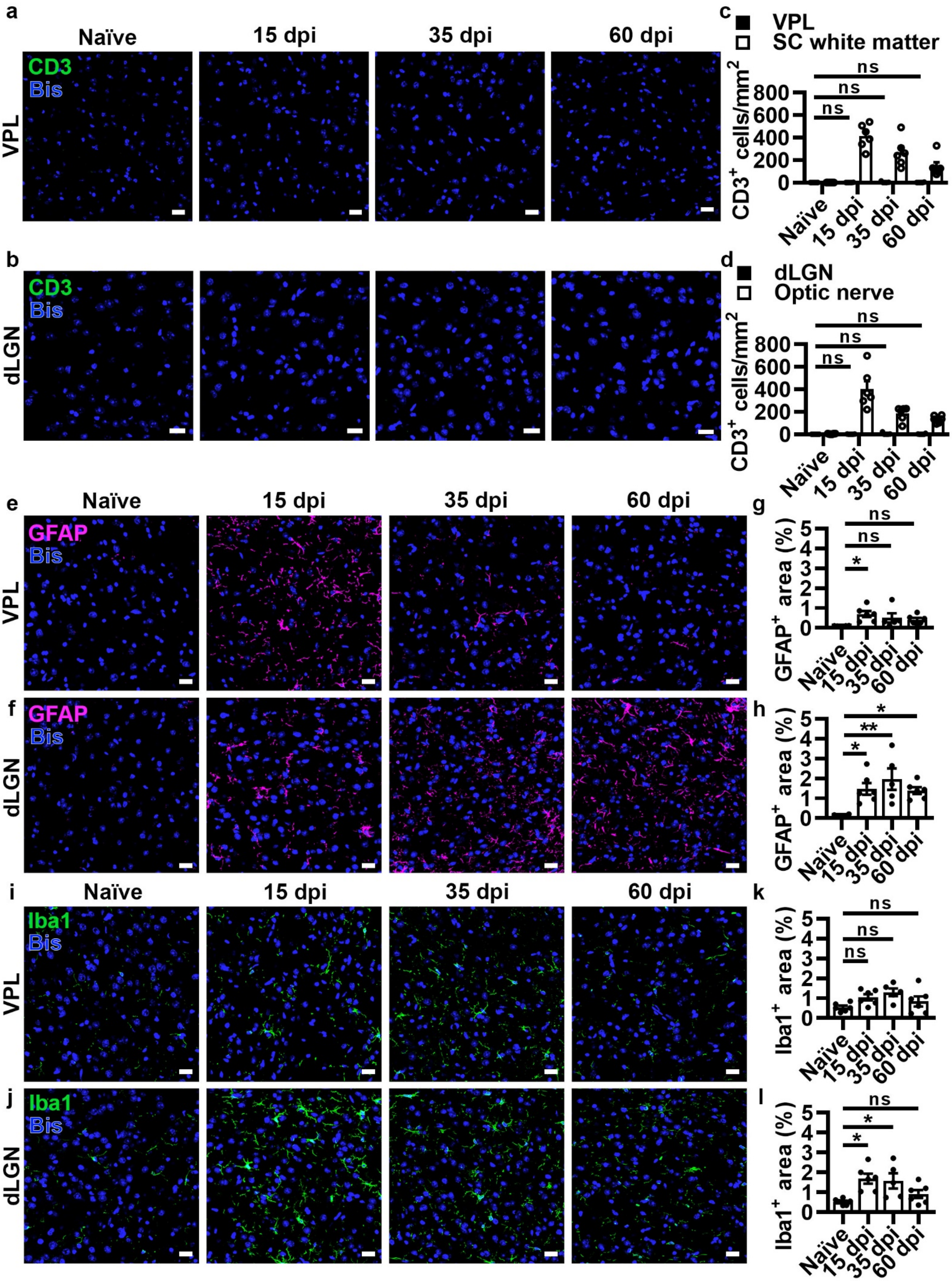

a
Naïve
15 dpi
35 dpi
60 dpi
CD3
Bis
VPL
b
CD3
Bis
dLGN
c
VPL
SC white matter
CD3+ cells/mm2
ns
ns
ns
Naïve
15 dpi
35 dpi
60 dpi
d
dLGN
Optic nerve
e
GFAP
Bis
VPL
f
GFAP
Bis
dLGN
g
GFAP+ area (%)
h
i
Iba1
Bis
VPL
j
Iba1
Bis
dLGN
k
Iba1+ area (%)
l

Figure S7. Reactive gliosis in the absence of $CD3^+$ T cell infiltration in the dLGN during EAE. *P* values listed consecutively are naïve vs 15, 35, or 60 dpi, respectively. (**a-b**) Representative images of $CD3^+$ T cells in the VPL and dLGN. Scale bars = 20 µm. (**c-d**) Quantification of $CD3^+$ T cells in the VPL relative to SC white matter (**c**, VPL compared to naïve: $P = 0.9619$, $P = 0.4423$, $P = 0.9619$; VPL compared to SC white matter naïve: $P > 0.9999$, 15 dpi: $P < 0.0001$, 35 dpi: $P < 0.0001$, 60 dpi: $P = 0.0127$) and $CD3^+$ T cells in the dLGN relative to ON (**d**, dLGN compared to naïve: $P = 0.9298$, $P = 0.3971$, $P = 0.8799$; dLGN compared to optic nerve naïve: $P > 0.9999$, 15 dpi: $P < 0.0001$, 35 dpi: $P = 0.0005$, 60 dpi: $P = 0.0176$). (**e-f**) Representative images of GFAP immunostaining in the VPL and dLGN. Scale bars = 20 µm. (**g-h**) Quantification of $GFAP^+$ area (**g**, VPL: *$P = 0.0299$, $P = 0.2383$, $P = 0.3819$; **h**, dLGN: *$P = 0.0165$, **$P = 0.0020$, *$P = 0.0214$). (**i-j**) Representative images of Iba1 immunostaining in the VPL and dLGN. Scale bars = 20 µm**.** (**k-l**) Quantification of $Iba1^+$ area (**k**, VPL: $P = 0.2551$, $P = 0.0571$, $P = 0.5712$; **l**, dLGN: *$P = 0.0123$, *$P = 0.0321$, $P = 0.4276$). Statistical differences were determined by one-way ANOVA with Holm-Šidák post-test. All data are expressed as means ± SEM including n = 5-6 mice per group, 3-6 fields from 2-3 sections for VPL and 6-12 fields from 2-3 sections for dLGN per mouse. Bis = bisbenzimide.

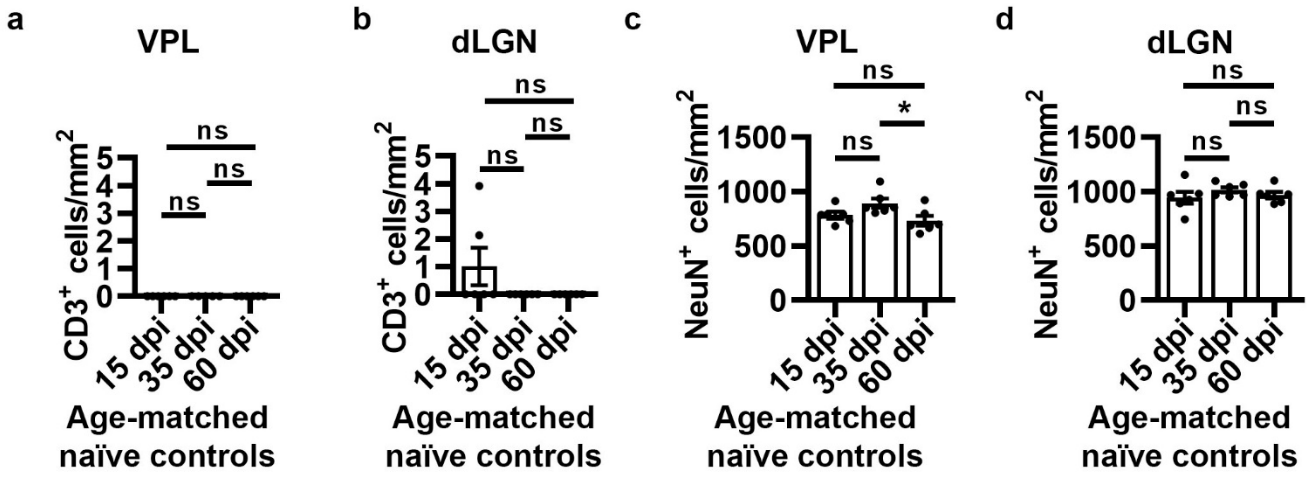

Figure S8. Quantification of CD3$^+$ T cells and NeuN$^+$ cells in age-matched naïve control groups in the dLGN and VPL. (**a-b**) Quantification of CD3$^+$ cells in age-matched naïve control groups (**a**, VPL: all naïve animals had a total of "0" T cells counted across all fields; **b**, dLGN: naïve for 15 vs 35 dpi $P = 0.2426$, 15 vs 60 dpi $P = 0.2426$, and 35 vs 60 dpi $P > 0.9999$). (**c-d**) Quantification of NeuN$^+$ cells in age-matched naïve control groups (**c**, VPL: naïve for 15 vs 35 dpi $P = 0.1425$, 15 vs 60 dpi $P = 0.3844$, and 35 vs 60 dpi $^*P = 0.0385$; **d**, dLGN: naïve for 15 vs 35 dpi $P = 0.5093$, 15 vs 60 dpi $P = 0.6826$, and 35 vs 60 dpi $P = 0.6255$). Statistical differences were determined by one-way ANOVA with Holm-Šidák post-hoc test. All data are expressed as means ± SEM including $n = 6$ mice per group, 3 to 6 fields from 2 to 3 sections per mouse.

References

Acknowledgements: This study was supported by the National Science Foundation 1648822, the National Eye Institute R01EY025687, R01EY032342, P30EY025585, T32 EY024236, the National Multiple Sclerosis Society FG-1807-31882, FG-2108-38411, the National Institute for Neurological Disorders K23NS109328, and an unrestricted award from Research to Prevent Blindness to the Department of Ophthalmology, Cleveland Clinic Lerner College of Medicine of Case Western Reserve University.
